# Executive functions in trauma-exposed youth: a meta-analysis

**DOI:** 10.1080/20008198.2018.1450595

**Published:** 2018-05-03

**Authors:** Rosanne Op den Kelder, Alithe L. Van den Akker, Hilde M. Geurts, Ramón J. L. Lindauer, Geertjan Overbeek

**Affiliations:** a Research Institute of Child Development and Education, University of Amsterdam, Amsterdam, The Netherlands; b De Bascule Academic Center for Child and Adolescent Psychiatry, Amsterdam, The Netherlands; c Research Institute of Child Development and Education/Research Priority Area YIELD, University of Amsterdam, Amsterdam, The Netherlands; d Department of Psychology (Brain & Cognition)/Research Priority Area YIELD, University of Amsterdam, Amsterdam, The Netherlands; e Dr. Leo Kannerhuis, Autism Clinic, Doorwerth, The Netherlands; f Department of Child and Adolescent Psychiatry, Academic Medical Center, University of Amsterdam, Amsterdam, The Netherlands

**Keywords:** Psychotrauma, meta-analysis, youth, executive functions, working memory, inhibition, cognitive flexibility, post-traumatic stress disorder, psicotrauma, metaanálisis, juventud, funciones ejecutivas, memoria de trabajo, inhibición, flexibilidad cognitiva, trastorno de estrés postraumático, 心理创伤, 元分析, 青年, 执行功能, 工作记忆, 抑制, 认知灵活性, 创伤后应激障碍, • Abusedviolence-exposedadopted and foster care youth have lower levels of inhibition.• Adopted and foster care youth have lower levels of cognitive flexibility.• Low-quality measurement studies show lower levels of working memory in trauma-exposed youth.• Executive functioning should be a focus in treatment of trauma-exposed youth.

## Abstract

An earlier meta-analysis and review indicated that trauma exposure may be related to lower levels of executive functioning in youth. Since different developmental trajectories were found for three core executive functions, the present study focused on working memory, inhibition, and cognitive flexibility specifically. We conducted a multi-level meta-analysis on 55 studies and 322 effect sizes published between 2001 and 2017 that were retrieved from MEDLINE, Embase, and PsycINFO. The 8070 participants in selected studies were aged 2–25 years. We investigated whether the association between constructs would be moderated by trauma-specific moderators (onset, duration, and type), and study (age, gender, ethnicity, and socio-economic status) and measurement (quality) characteristics. We found small to medium effect sizes for working memory (*d *= −0.49), inhibition (*d *= −0.46), and cognitive flexibility (*d *= −0.44). Moderator analyses showed that, for working memory, when studies used low-quality measurements the effect size was significantly stronger than when studies used high-quality measurements.Compared to single trauma-exposed youth, violence-exposed/abused and foster care/adopted youth showed more problems in inhibition, and foster care/adopted youth showed more problems in cognitive flexibility. Our findings imply that trauma-exposed youth have lower levels of executive functions. Clinical practice should incorporate problems in executive functioning, especially working memory, inhibition, and cognitive flexibility, in assessment and treatment guidelines.

## Abstract (es)

**Planteamiento:** Un metanálisis y una revisión previos indicaron que la exposición al trauma podría estar relacionada con niveles más bajos de funcionamiento ejecutivo en los jóvenes.

**Objetivo:** A medida que se encontraron diferentes trayectorias de desarrollo para tres funciones ejecutivas centrales, nos enfocamos específicamente en la memoria de trabajo, la inhibición y la flexibilidad cognitiva.

**Método:** Realizamos un metanálisis multinivel con 55 estudios y 322 tamaños de efectos publicados entre 2001 y 2017, obtenidos de MEDLINE, Embase y PsycINFO. Los 8070 participantes en los estudios seleccionados tenían entre 2 y 25 años. Investigamos si la asociación entre los constructos sería moderada por los moderadores específicos del trauma (inicio, duración y tipo), y las características del estudio (edad, género, etnia, estatus socioeconómico) y características de medición (calidad).

**Resultados:** Encontramos tamaños de efecto de pequeño a mediano para la memoria de trabajo (d = −0.49), la inhibición (d = −0.46) y la flexibilidad cognitiva (d = −0.44). Los análisis del moderador mostraron que, para la memoria de trabajo, cuando los estudios utilizaron mediciones de baja calidad, el tamaño del efecto fue significativamente más fuerte que cuando los estudios utilizaron mediciones de alta calidad. En comparación con los jóvenes expuestos a un solo trauma, los jóvenes expuestos a violencia /abuso y los jóvenes que viven en condiciones de acogida o han sido adoptados mostraron más problemas en la inhibición y los jóvenes que viven en condi- ciones de acogida o han sido adoptados mostraron más problemas de flexibilidad cognitiva.

**Conclusión:** Nuestros hallazgos implican que los jóvenes expuestos a trauma tienen niveles más bajos de funciones ejecutivas. En sus guías de evaluación y tratamiento, la práctica clínica debe incorporar problemas en el funcionamiento ejecutivo, especialmente la memoria de trabajo, la inhibición y la flexibilidad cognitiva.

## 创伤暴露青少年的执行功能：一个元分析

**背景**：一个早期的元分析和综述指出创伤暴露可能和青少年的执行功能较弱有关。

**目的：**由于三个核心执行功能有不同的发展轨迹，我们的研究分别集中在工作记忆、抑 制功能和认知灵活性上。

**方法：**我们在55个研究和322个效应量上使用了多层元分析，这些研究发表于2001-2017之 间，来自于 MEDLINE, Embase和PsycINFO。在被选择的研究中，8070名被试的年龄范围在2- 25岁。我们考察了这些结构之间的联系是否会被创伤特异的调节变量（发病、持续时间、 类型），研究性质（年龄、性别、民族、社会经济地位）和测量特征（质量）所影响。

**结果：**我们发现对工作记忆（d = −0.49），抑制（d = −0.46）和认知灵活性（d = −0.44）但是调节分析显示对工作记忆来说，当研究使用低质量测量时，效应大小显着强于当研究使用高质量测量时。相比于单一创伤暴露青少年，暴露于暴 力/虐待和领养寄养的青少年表现出抑制功能的问题，领养寄养青少年表现出更多认知灵 活性的问题。

**结论：**我们研究发现暗示，创伤暴露青少年的执行功能更弱。临床实践应该也在评估和 治疗指导手册中也包含执行功能的问题，特别是工作记忆，抑制和认知灵活性。

## 1. Introduction

Many children and adolescents, approximately between 25% and 66%, are exposed to traumatic events during childhood (Copeland, Keeler, Angold, & Costello, 2007; Costello, Erkanli, Fairbank, & Angold, 2002). Trauma-exposed youth have a wide array of emotional and physical health problems. Previous meta-analyses showed that trauma exposure is associated with post-traumatic stress complaints, internalizing and externalizing problems (Fowler, Tompsett, Braciszewski, Jacques-Tiura, & Baltes, 2009), depression, suicide attempts, drug use, sexually transmitted diseases (Norman et al., 2012), and various physical health problems such as neurological, musculoskeletal, respiratory, cardiovascular, and metabolic problems (Wegman & Stetler, 2009). Besides these emotional and physical consequences of trauma exposure, results of previous reviews showed that cognitive functioning, more specifically executive functioning, is also affected by early life stress and trauma exposure in youth (Kavanaugh, Dupont-frechette, Jerskey, & Karen, 2017; Malarbi, Abu-Rayya, Muscara, & Stargatt, 2017). Whereas earlier research focused on the impact of trauma and maltreatment on overall executive skills in youth, we distinguish three core executive functions: working memory, inhibition, and cognitive flexibility.

### 1.1. Trauma exposure and executive functions

Executive functions cover multiple skills, such as inhibition, organization, cognitive flexibility, self-monitoring, regulation of emotions, working memory, and attention. These are essential in preparing and executing goal-directed behaviour (Diamond, 2013; Goldstein, Naglieri, Princiotta, & Otero, 2014). Most studies indicate that executive function processes in youth are distinct, albeit moderately associated with each other (Best, Miller, & Jones, 2009; Miyake et al., 2000). Some debate exists on whether separate executive functions can be subsumed in a single, central executive function. However, impairment in global executive functioning is rare. Different regions of the prefrontal cortex are activated in different executive function tasks, and distinct developmental pathways have been identified for different executive processes (Anderson, 2002; Best et al., 2009). Most empirical neuropsychological research differentiates between three core executive functions: inhibition, working memory, and cognitive flexibility. These three domains are considered core executive functions from which higher order functions such as reasoning, problem solving, and planning arise (e.g. Diamond, 2013; Miyake et al., 2000). Therefore, in this study we focus on working memory, inhibition, and cognitive flexibility.

The first core executive function, working memory, is a cognitive process of temporarily storing and manipulating information. Working memory is distinct from short-term memory, because short-term memory only stores information, without manipulating it (Baddeley, 2012; Goldstein et al., 2014). Verbal working memory (which ‘works’ with words, numbers, and letters) and visuospatial working memory (which ‘works’ with figures and spatial information) are commonly distinguished. Inhibition or inhibitory control, the second core executive function, refers to the ability to control attention, thoughts, and emotions, thereby suppressing dominant, automatic, or prepotent responses when necessary (Diamond, 2013; Miyake et al., 2000). Prepotent response inhibition and interference control are commonly distinguished aspects of inhibition (Friedman & Miyake, 2004; Miyake & Friedman, 2012). Prepotent response inhibition enables us to suppress a dominant motor response (Aron, 2011; Miyake et al., 2000), whereas interference control is the ability to ignore irrelevant information by resisting distractor interference (Friedman & Miyake, 2004; Nigg, 2000). The third core executive function, cognitive flexibility, refers to the ability to switch between tasks, demands, priorities, rules, and perspectives. It helps in thinking ‘outside the box’ and forming creative solutions (Best et al., 2009; Diamond, 2013). Being cognitively flexible enables learning from mistakes and generating alternative solutions. Inflexible individuals fail to adapt to new situations or demands; they continue making the same mistakes, showing rigid and ritualistic behaviour (Anderson, 2002).

A previous meta-analysis and a review showed that trauma-exposed and maltreated youth performed worse on executive functions than controls (Kavanaugh et al., 2017; Malarbi et al., 2017). Trauma exposure is thought to influence executive functions by impacting underlying neurobiological mechanisms. As brain development continues into adulthood, trauma exposure may impact the development of executive functions in youth. Empirical research in humans showed that early life stress such as maltreatment affects the hypothalamic–pituitary–adrenocortical axis, but also structures of the corticolimbic networks (De Bellis, 2001; De Bellis et al., 1999; Gunnar & Quevedo, 2007). Most affected brain regions in maltreated youth are the prefrontal cortex, orbitofrontal cortex, anterior cingulate cortex, and amygdala (Cowell, Cicchetti, Rogosch, & Toth, 2015; De Bellis & Thomas, 2003; Teicher & Samson, 2016). Atypicalities in structural connectivity between the anterior cingulate cortex and dorsolateral, orbitofrontal, and ventromedial prefrontal cortices are shown by brain imaging studies (Hart & Rubia, 2012). These brain networks are activated during response inhibition, working memory, and emotion processing tasks, which suggest that the neural networks for executive functioning are affected by trauma exposure in youth (Teicher & Samson, 2016).

Development of executive functions continues until young adulthood, with the most rapid development taking place during preschool and the early school years (Best & Miller, 2010; Friedman et al., 2015; Miyake & Friedman, 2012). However, the separate executive functions show slightly different developmental trajectories (Best & Miller, 2010; Huizinga, Dolan, & van der Molen, 2006). Working memory seems to follow a linear development from preschool to adolescence. Inhibition, on the other hand, improves most rapidly during the preschool years, followed by a modest linear improvement through adolescence. For cognitive flexibility, preschoolers are able to handle shifts of simple tasks and this increases during childhood to more unexpected shifts between complex tasks. Switching of complex tasks seems to mature by middle adolescence. All executive function skills show a developmental pattern of ‘rises and falls’, which is related to brain development (Best & Miller, 2010; Johnson & De Haan, 2011). These different developmental trajectories may suggest different effects of both timing and the duration of trauma exposure (Teicher & Samson, 2016) on executive functions.

### 1.2. Moderators

By performing moderator analyses, we can examine the influence of trauma-specific moderators, sample characteristics, and executive function task characteristics on the strength of the association between trauma exposure and executive functions. First, we tested whether trauma characteristics (i.e. type, onset, duration, and post-traumatic stress complaints) influenced the strength of the association between exposure and executive functions. Specifically, interpersonal, repeated trauma has more severe effects on the brain than single trauma. The earlier and the more prolonged the trauma exposure has been, the stronger the impact of trauma exposure is (e.g. Cook et al., 2005; Bruce et al., 2014; Cowell et al., 2015; Teicher & Samson, 2016). Consequently, we tested whether earlier onset and longer duration of trauma, trauma subtype (single trauma, violence/abuse, adoption/foster care), and post-traumatic stress disorder (PTSD) would be associated with significantly lower executive functions.

Sample characteristics (age, socio-economic status, gender, and ethnicity) could influence the strength of the association between trauma exposure and executive function in youth. Differential effects of trauma exposure have been established for gender (Alisic et al., 2014), age (e.g. Lupien, McEwen, Gunnar, & Heim, 2009; Weems et al., 2010), and ethnicity (López et al., 2017), with stronger effects of trauma exposure for girls, younger children, and Hispanic and black adolescents.

The strength of the association between trauma exposure and executive functions could also be influenced by the quality of the executive function measure. Working memory, inhibition, and cognitive flexibility are moderately associated (Best et al., 2009; Miyake & Friedman, 2012), complicating the clear assessment of executive functions (Diamond, 2013). For example, tasks such as the Digit Span, go/no-go tasks, and the Wisconsin Card Sorting Task have various outcome measures. These outcome measures vary in how purely they assess the different executive functions (Huizinga et al., 2006). Therefore, we tested whether the quality of the outcome measurement influences the strength of the association of trauma exposure with executive functions in youth.

In sum, we investigated whether trauma-specific characteristics (onset, duration, type, and PTSD complaints), sample characteristics (gender, age, and ethnicity), and executive function task characteristics (executive function measure) influenced the relationship between trauma exposure and executive functions in youth.

### 1.3. The present study

As our understanding of the mental health consequences of trauma exposure in youth has increased considerably (e.g. Alisic, Jongmans, van Wesel, & Kleber, 2011; Jonkman, Verlinden, Bolle, Boer, & Lindauer, 2013; Lamers-Winkelman, Willemen, & Visser, 2012), treatments for youth have been developed to treat these (Morina, Koerssen, & Pollet, 2016). However, the link between executive functions and trauma exposure in youth is less well understood. Only the Attachment, Regulation, and Competence model includes executive functions in its guidelines (Blaustein & Kinniburgh, 2015). Our aim is to inform clinical practice to allow for integration of executive functions in therapy protocols for traumatized youth. Therefore, we investigated the extent to which youth exposed to trauma suffer from problems with their executive functions. In addition, we investigated whether different moderators influence the strength of the relationship between trauma exposure and executive functions. To answer these questions, we conducted what is, to our knowledge, the first multi-level meta-analysis to investigate working memory, inhibition, and cognitive flexibility in trauma-exposed children and adolescents.

## 2. Methods

### 2.1. Selection of studies

This analysis included: (1) studies comparing working memory, inhibition, and/or cognitive flexibility between trauma exposed and non-exposed individuals, and studies that reported a correlation coefficient to assess the relationship between trauma exposure and these executive functions; (2) studies reported in English; and (4) studies with samples aged between 0 and 25 years old. We focused on this specific age range because of strong indications that the development of the prefrontal cortex is largely accomplished by around the age of 25 years (e.g. Arain et al., 2013). Exclusion criteria were: studies including participants with traumatic brain injury and current drug abuse, as these factors are known to influence executive functioning (Fernández-Serrano, Pérez-García, Schmidt Río-Valle, & Verdejo-García, 2010; Gioia, Isquith, Kenworthy, & Barton, 2002); studies that examined foster care or adopted youth but had no control group, as traumatic exposure varies widely in these samples and drawing conclusions is problematic without a reference group. Primary outcome measures pertained to working memory, cognitive flexibility, and inhibitory control. Trauma exposure was defined as exposure to events that, according to the Diagnostic and Statistical Manual of Mental Disorders, Fifth Edition (DSM-5) (American Psychiatric Association, 2013), are considered potentially traumatic. For example, a traffic accident, witnessing domestic violence or a shooting, living in a war environment, and neglect are considered traumatic events (American Psychiatric Association, 2013).

### 2.2. Information sources

The search covered PsycINFO, Embase, and MEDLINE (until August 2017), and was based on the Meta-Analysis Reporting Standards. Appendix A shows the full electronic search strategy.

### 2.3. Study selection

**Figure 1.**
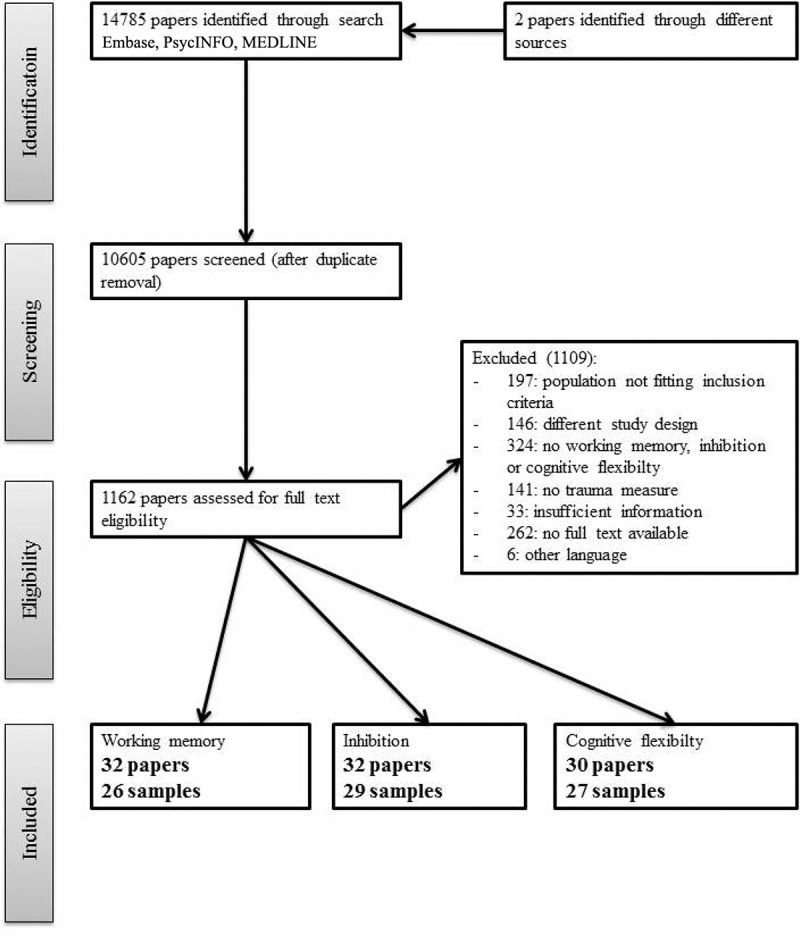
Preferred Reporting Items for Systematic Reviews and Meta-Analyses (PRISMA) overview for eligibility assessment.

The eligibility assessment is displayed in Figure 1, and was performed by two independent reviewers in a standardized manner (see Appendix B). In the title and abstract screening phase, 1000 of the 10,605 papers were screened by two reviewers (first author and screener 1), and disagreements were resolved by consensus. In the second screening phase, full text screening, 1162 papers were screened by two reviewers (screeners 1 and 2). Disagreements were resolved by consultation with the first author. Finally, we included 32, 32, and 30 papers on working memory, inhibition, and cognitive flexibility, respectively.

### 2.4. Data collection process

We developed a data-extraction sheet (Appendix C). The first author coded all studies, and the second author coded 15%, and disagreements were resolved by discussion. Interrater agreement was 1.00 for Cohen’s kappa and intraclass correlation ranged between 0.96 and 1.00. Of 64 authors contacted for further information, 15 responded and 12 provided data that were requested. We could not retrieve the full text for 262 papers. After further enquiries with authors we retrieved an additional 13 full text papers. However, none of these papers was eligible for inclusion. References for the included papers are listed in Appendix E.

### 2.5. Data items

Information was extracted from each included study on: (1) characteristics of participants (i.e. age, gender, socio-economic status, years of schooling, ethnicity); (2) study characteristics (i.e. research design, publication status, and overall study quality); (3) type of trauma exposure (i.e. trauma type, onset, and duration); (4) post-traumatic stress (post-traumatic stress complaints, PTSD diagnosis); and (5) type of outcome measure (e.g. Wechsler Intelligence Scale for Children Digit Span backwards, Trail Making Test-B).

For the participant characteristics, overall study quality (at study level) was assessed by two independent research assistants. We used the Quality Assessment Tool for Quantitative Studies of the Effective Public Health Practice Project (Thomas, Ciliska, Dobbins, & Micucci, 2004). This is an assessment tool for the quality of both randomized and case–control studies. A global quality rating of weak, moderate, or strong was assigned by both reviewers. There was 97.8% consensus between the two reviewers. Furthermore, discrepancies were at subscale level, not at the global rating level.

For trauma characteristics, type of trauma exposure was divided into three categories: single trauma exposure; exposure to violence, abuse, or neglect; and adopted or foster care youth. Onset and duration of trauma exposure were measured using reported information about the mean age of the start of trauma exposure and the mean duration (in years). See Appendix C for more detailed information about data extraction.

For studies reporting on working memory, inhibition, and/or cognitive flexibility, we coded type of outcome measure for each effect size in all data sets. With regard to the outcome measure used, we coded quality of the measurement instrument, based on the extent to which measurement of cognitive flexibility, inhibition, and working memory were confounded with the assessment of speed or other executive function elements and the level of cognitive load of the measures. These decision rules were based on the executive function research expertise of the third author and conform to recent literature specifications about quality of outcome measures of executive function (e.g. Tamminga, Reneman, Huizenga, & Geurts, 2016). The codes are described in Appendix D.

### 2.6. Strategy of analysis

In 65.5%, 68.8%, and 73.% of the papers about respectively working memory, inhibition, and cognitive flexibility, more than one relevant effect size was reported. Papers reported on multiple effect sizes for the following reasons: (1) different outcome measures were used to assess executive functions; (2) different aspects of executive functions were measured (e.g. verbal versus non-verbal working memory); (3) various assessments of the association between trauma exposure and executive functions in time were included; and (4) different groups were investigated to assess the association between trauma exposure and executive functions (e.g. comparisons between maltreated children with PTSD and a control group, and comparisons between maltreated children without PTSD and a control group). Cohen’s *d* was calculated using reported means and standard deviations, and reported correlations were transformed to Cohen’s *d*. The SPSS syntax for effect size calculation was double-checked by the second author.

We used a three-level meta-analytic random effects model as it increases power (Assink & Wibbelink, 2016). It gives us more information because effect sizes are not eliminated or averaged (Assink & Wibbelink, 2016; Cheung, 2014). We modelled three levels of variance: (1) variance in effect sizes due to random sampling; (2) variance in effect size due to differences within studies; and (3) variance in effect sizes between studies (Borenstein, Hedges, Higgins, & Rothstein, 2010). This multi-level approach allows dependency of effect sizes within studies. As a result, we can include multiple effect sizes per study and test whether there are between- or within-study differences in effect sizes when heterogeneity is assumed (Assink & Wibbelink, 2016). Moderator analyses can explain within- or between-study differences in effect sizes when there is heterogeneity (Borenstein et al., 2010). We used an expert tutorial (Assink & Wibbelink, 2016) for the software R to perform statistical analyses for our three-level meta-analyses with a random model using the Metafor package (Viechtbauer, 2006).

### 2.7. Publication bias

Publication selection bias is a common issue in meta-analyses (Borenstein et al., 2010). We used the PET-PEESE approach to investigate publication selection bias, as this approach has been shown to outperform the Fail Safe *N* analysis and Trim & Fill strategy (Stanley & Doucouliagos, 2014). The PET-PEESE approach consists of two steps. The first step, the precision-effect-test (PET), is based on results on the Egger test, an analysis in which the standard error is used as a moderator. When the intercept in this model is not significantly different from zero, a significant moderator implicates possible publication bias. When the intercept is significantly different from zero, we take the next step: PEESE (precision-effect estimate with standard error). However, instead of the standard error, the variance is included as a moderator. When the effect size varies significantly with the standard error, the analysis gives an implication for publication bias. However, it should be noted that all publication bias analyses have a low power to detect bias (Borenstein et al., 2010; Stanley & Doucouliagos, 2014). Furthermore, we used the PET-PEESE approach in a random model but, as in all other publication bias assessments, it is designed for a fixed effects model.

## 3. Results

### 3.1. Associations between trauma exposure and executive functions

**Figure 2.**
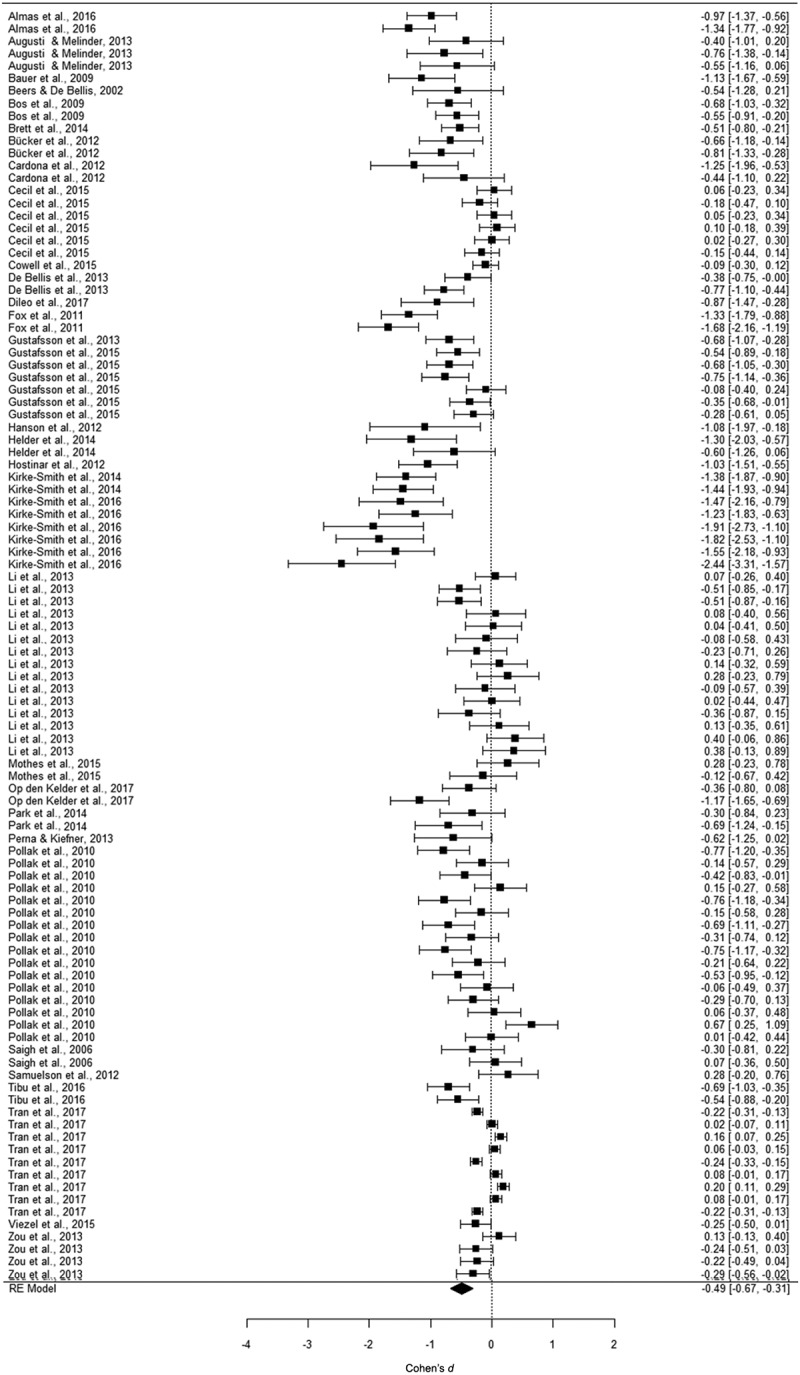
Forest plot of the meta-analysis on the association between trauma exposure and working memory. RE, random effects.

**Figure 3.**
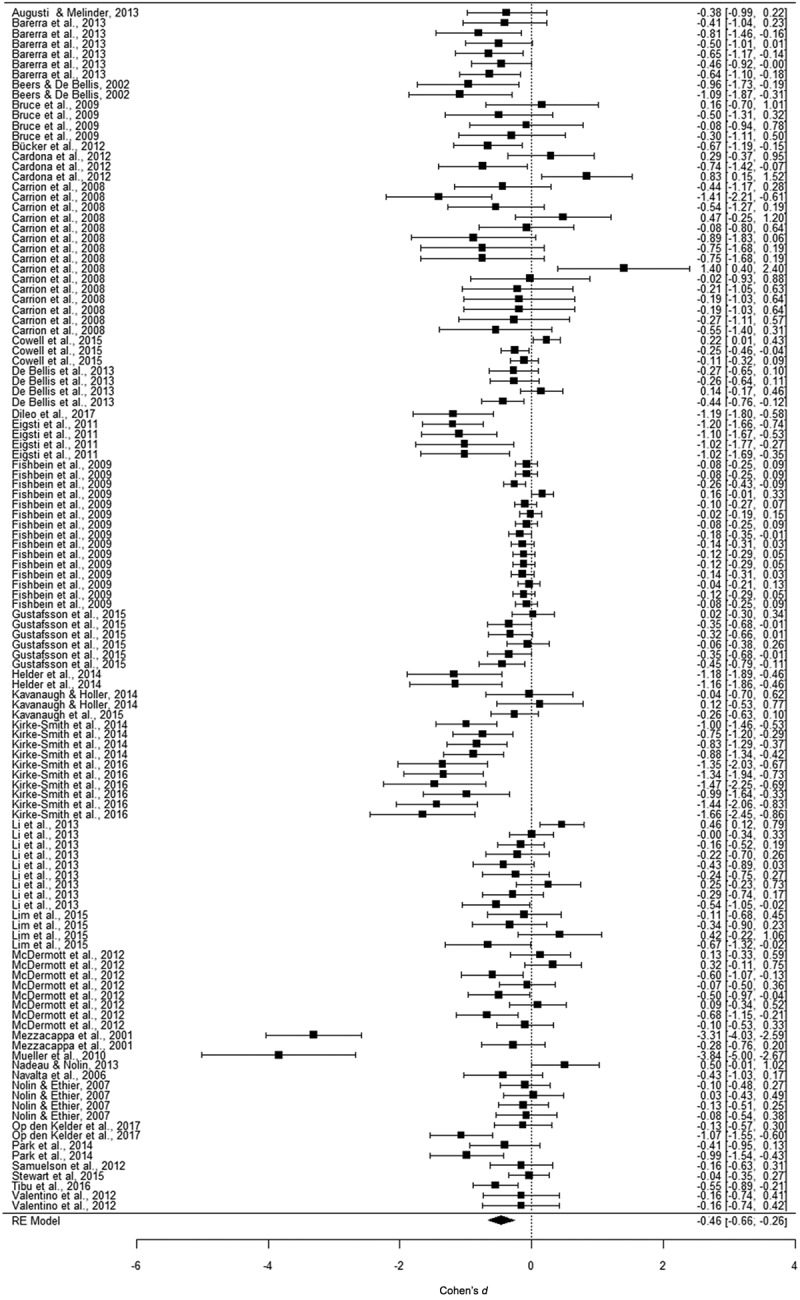
Forest plot of the meta-analysis on the association between trauma exposure and inhibition. RE, random effects.

**Figure 4.**
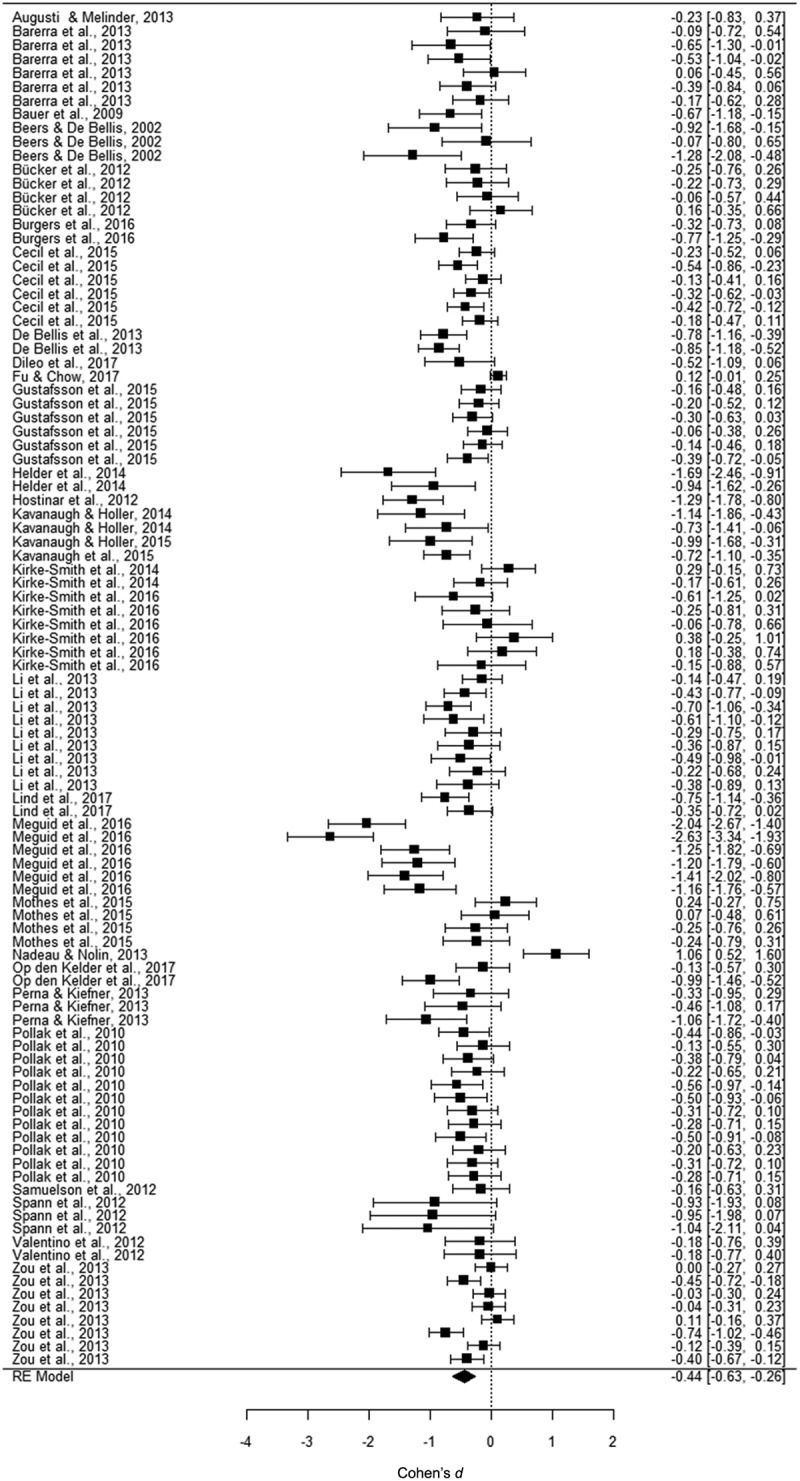
Forest plot of the meta-analysis on the association between trauma exposure and cognitive flexibility. RE, random effects.

We performed three separate multi-level meta-analyses. Overall effect sizes are displayed in Table 1. For working memory, we examined 26 samples and 102 effect sizes, reporting data on 5172 participants aged between 3 and 24 years. Figure 2 displays a forest plot showing the effect sizes and their confidence intervals. The analysis yielded a significant, small to medium effect size of *d *=* −*0.49 in a random model. This indicated that trauma-exposed youth perform worse on working memory than non-exposed youth. For inhibition, we examined 29 samples with 119 effect sizes, reporting data on 3391 participants aged between 5 and 20 years. In Figure 3, effect sizes and their confidence intervals are displayed. The analysis yielded a significant, small to medium effect size of *d *= −0.46 in a random model. Thus, trauma-exposed youth also perform worse on inhibition tasks than non-exposed youth. For cognitive flexibility, we examined 27 samples with 101 effect sizes, reporting data on 2959 participants aged between 2 and 24 years. In Figure 4, the forest plot displays the effect sizes and confidence intervals. This analysis yielded also a significant, small to medium effect size of *d = −*0.44 in a random model. When investigating outliers for the variables of interest, we found four outliers in the effect sizes: working memory (one outlier), inhibition (two outliers), and cognitive flexibility (one outlier). After trimming these outliers to the value of the highest/lowest effect size plus/minus one unit, we found that the mean effect size, although still significant, decreased to −0.37 for inhibition, but remained the same for working memory and cognitive flexibility.

**Table 1.** Effect sizes (ES) and confidence intervals (CI) for meta-analyses on the association between trauma exposure and working memory, inhibition, and cognitive flexibility.

	*K*	ES	*n*	*d*	95% CI	*p*
Working memory	26	102	5172	−0.49	−0.67 ; −0.31	< 0.001
Inhibition	29	119	3391	−0.46	−0.66 ; −0.26	< 0.001
Cognitive flexibility	27	101	2959	−0.44	−0.63 ; −0.26	< 0.001

*K* = number of samples.

### 3.2. Variation in effect sizes

To investigate whether moderator analyses were necessary, we analysed whether variation in effect sizes could be attributed to random sampling error, within-study variance (level 2), or between-study variance (level 3). For working memory, effect sizes were heterogeneous as both within-study variance (*σ*^2^_v_ = 0.05), *X*^2^ (1) = 105.64, *p* < 0.001), and between-study variance were significant (*σ*^2^_v_ = 0.16, *X*^2^ (1) = 69.00, *p* < 0.001). Of the total variance, 20.4% was attributable to within-study differences and 72.0% to between-study differences. For inhibition, both within-study variance (*σ*^2^_v_ = 0.04), *X*^2^ (1) = 17.11, *p *< 0.001) and between-study variance were significant (*σ*^2^_v_ = 0.23, *X*^2^ (1) = 45.32, *p* < 0.001). Of the total variance, 13.3% was attributable to within-study differences and 76.4% to between-study differences. When we analysed the heterogeneity of effect sizes for cognitive flexibility, we found significant within-study variance (*σ*^2^_v_ = 0.02), *X*^2^ (1) = 7.20, *p* = 0.007) and between-study variance (*σ*^2^_v_ = 0.19, *X*^2^ (1) = 54.02, *p* < 0.001). Of the total variance, 7.2% was attributable to within-study differences and 75.6% to between-study differences. In sum, significant heterogeneity was found between and within studies for working memory, inhibition, and cognitive flexibility. To explain the variation in effect sizes on the second and third levels, we added moderators to the random effects model.

### 3.3. Moderator analyses

We examined the extent to which moderators influenced the association between trauma exposure and executive functions by adding moderators as covariates (separately) to the random effect models. Table 2 displays the results of these analyses for working memory. We found that the quality of the measurement instrument (*F* (2,99) = 6.50, *p* = 0.002) influenced the association between trauma exposure and working memory significantly. The mean effect size for low-quality measurements was significantly  stronger than the effect size that was found for high-quality measurements. We found that study quality was not an overall significant moderator (*F* (2,99) = 2.43, *p* = 0.093). However, we found that studies with a weak quality had a mean effect size that was significantly stronger than studies with a strong quality.

**Table 2.** Moderator analyses for the association between trauma exposure and working memory.

Variable	*K*	ES	*β*_0_ (SE)	*t*_0_	*β*_1_ (SE)	*t*_1_	*F* (df_1_,df_2_)
Study characteristics							
Age (mean centred)	24	94	−0.47 (0.82)	−5.74***	0.04 (0.02)	1.85	3.42 (1,92)
Gender (% female, mean centred)	25	90	−0.45 (0.09)	−5.23***	−0.00 (0.00)	0.74	0.55 (1,88)
Ethnicity (% minority, mean centred)	11	53	−0.32 (0.09)	−3.41**	0.00 (0.00)	1.02	1.03 (1,51)
Socio-economic status (SES)	26	102					0.05 (1,100)
Not controlled for SES (RC)	15	65	−0.51 (0.12)	−4.18***			
Controlled for SES	11	37			0.04 (0.19)	0.218	
Study quality	26	102					2.43 (2,99)
Strong (RC)	9	25	−0.34 (0.14)_a_	−2.49*			
Moderate	14	47	−0.52 (0.12)_ab_	−4.38***	−0.18 (0.16)	−1.15	
Weak	7	30	−0.67 (0.14)_b_	−4.98***	−0.34 (0.15)	−2.20*	
Trauma characteristics							
Onset	6	11	−0.72 (0.29)	−2.48*	−0.01 (0.07)	0.17	0.03 (1,9)
Duration	6	17	−0.74 (0.27)	−2.78*	−0.01 (0.08)	−0.17	0.03 (1,15)
Type	25	101					1.73 (2,98)
Single (RC)	4	10	−0.28 (0.16)_a_	−1.71			
Violence/abuse	16	59	−0.41 (0.11)_a_	−3.79***	−0.14 (0.14)	−0.95	
Adoption/foster care	7	32	−0.71 (0.17)_a_	−4.11***	−0.44 (0.24)	−1.86	
PTSD diagnoses	7	26					3.47 (1,24)
No diagnoses in sample (RC)	4	13	−0.38 (0.18)	−2.10*			
Diagnoses in sample	7	13			−0.24 (0.13)	−1.86	
Measurement characteristics							
Quality	26	102					6.50 (2,99)*
High (RC)	8	28	−0.27 (0.12)_a_	−2.32*			
Medium	11	43	−0.44 (0.12)_ab_	−3.75***	−0.17 (0.11)	−1.62	
Low	14	31	−0.65 (0.12)_b_	−5.64***	−0.38 (0.11)	−3.54***	

*K* = number of samples; ES = number of effect sizes; *β*_0_ = mean effects size (Cohen’s *d*); *t*_0_ = test statistic for difference mean effect with zero; *β*_1_ = regression coefficient; *t*_1_ = test statistic of difference of mean effect size with the reference category (RC); *F* (df_1_,df_2_) = test statistic for testing significance of moderator; violence/abuse includes physical and emotional abuse, neglect, sexual abuse, and violence exposure; values with the same subscripts do not differ significantly from each other at *p* < 0.05.

*p < 0.05, **p < 0.01, ***p < 0.001.

For inhibition, only type of trauma exposure was a significant moderator (*F* (2,116) = 5.21, *p* = 0.007). The mean effect size for single trauma exposure did not differ significantly from zero. No significant differences were found between violence-exposed/abused and adopted/foster care youth, but the average effect sizes for these groups differed significantly from zero and from single trauma-exposed youth. Study quality was, overall, not a significant moderator (*F* (2,116) = 2.42, *p* = 0.092). However, studies with a moderate quality had a significantly stronger effect size than weak-quality studies. Results of moderator analyses are displayed in Table 3.

**Table 3.** Moderator analyses for the association between trauma exposure and inhibition.

Variable	*K*	ES	*β*_0_ (SE)	*t*_0_	*β*_1_ (SE)	*t*_1_	*F* (df_1_,df_2_)
Study characteristics							
Age (mean centred)	27	85	−0.49 (0.11)	−4.66***	0.02 (0.03)	−0.62	0.38 (1,83)
Gender (% female, mean centred)	28	109	−0.46 (0.10)	−4.47***	0.00 (0.00)	0.43	0.19 (1,107)
Ethnicity (% minority, mean centred)	14	48	−0.25 (0.08)	−3.20***	0.00 (0.00)	0.27	0.07 (1,46)
Socio-economic status (SES)	29	119					0.14 (1,117)
Not controlled for SES (RC)	17	83	−0.43 (0.13)	−3.26**			
Controlled for SES	12	36			−0.08 (0.21)	−0.37	
Study quality	29	119					2.43 (2,116)
Strong (RC)	11	37	−0.42 (0.15)_ab_	−2.85**			
Moderate	11	41	−0.64 (0.14)_a_	−4.48***	−0.22 (0.20)	−1.10	
Weak	10	41	−0.29 (0.14)_b_	−2.07*	0.14 (0.17)	0.83	
Trauma characteristics							
Onset	10	27	−1.02 (0.51)	−2.01	−0.09 (0.12)	−0.79	0.62 (1,25)
Duration	9	23	−1.13 (0.46)	−2.45*	−0.11 (0.10)	1.051	1.11 (1,21)
Type	29	119					5.21 (2,116)**
Single (RC)	3	6	0.04 (0.19)_a_	0.21			
Violence/abuse	22	90	−0.43 (0.12)_b_	−3.58***	−0.47 (0.16)	−2.85**	
Adoption/foster care	6	23	−0.79 (0.24)_b_	−3.31***	−0.83 (0.31)	−2.72**	
PTSD diagnoses	14	55					0.47 (1,53)
No diagnoses in sample (RC)	6	13	−0.48 (0.18)	−2.63*			
Diagnoses in sample	13	42			−0.09 (0.13)	−0.69	
Measurement characteristics							
Quality	29	119					0.04 (2,116)
High (RC)	15	43	−0.45 (0.11)_a_	−4.04***			
Medium	14	38	−0.46 (0.12)_a_	−4.03***	0.01 (0.09)	−0.14	
Low	11	38	−0.48 (0.12)_a_	−3.87***	0.03 (0.11)	−0.27	

*K* = number of samples; ES = number of effect sizes; *β*_0_ = mean effects size (Cohen’s *d*); *t*_0_ = test statistic for difference mean effect with zero; *β*_1_ = regression coefficient; *t*_1_ = test statistic of difference of mean effect size with the reference category (RC); *F* (df_1_,df_2_) = test statistic for testing significance of moderator; violence/abuse includes physical and emotional abuse, neglect, sexual abuse, and violence exposure; values with the same subscripts do not differ significantly from each other at *p* < 0.05.

*p < 0.05, **p < 0.01, ***p < 0.001.

**Table 4.** Moderator analyses for the association between trauma exposure and cognitive flexibility.

Variable	*K*	ES	*β*_0_ (SE)	*t*_0_	*β*_1_ (SE)	*t*_1_	*F* (df_1_,df_2_)
Study characteristics							
Age (mean centred)	26	89	−0.38 (0.08)	−4.68***	0.02 (0.02)	0.91	0.84 (1,87)
Gender (% female, mean centred)	25	85	−0.41 (0.08)	−4.88***	−0.00 (0.00)	−0.61	0.37 (1,83)
Ethnicity (% minority, mean centred)	14	43	−0.36 (0.12)	−3.10**	0.00 (0.00)	−1.65	2.73 (1,41)
Socio-economic status (SES)	27	101					0.00 (1,99)
Not controlled for SES (RC)	16	55	−0.45 (0.12)	−3.64***			
Controlled for SES	11	46			0.01 (0.19)	0.05	
Study quality	27	101					0.87 (2,98)
Strong (RC)	6	21	−0.33 (0.13)_a_	−2.49*			
Moderate	11	45	−0.57 (0.13)_a_	−4.33***	−0.23 (0.18)	−1.28	
Weak	12	35	−0.42 (0.15)_a_	−2.81**	−0.09 (0.18)	−0.48	
Trauma characteristics							
Onset	2	6	−0.15 (0.28)	0.54	−0.06 (0.09)	−0.71	0.50 (1,4)
Duration	4	13	−0.87 (0.85)	−1.02	−0.21 (0.36)	0.58	0.33 (1,11)
Type	27	101					2.62 (2,98)
Single (RC)	3	5	−0.17 (0.17)_a_	−1.01			
Violence/abuse	21	78	−0.39 (0.10)_ab_	−3.97***	−0.22 (0.15)	−1.49	
Adoption/foster care	5	18	−0.78 (0.21)_b_	−3.67***	−0.61 (0.27)	−2.25*	
PTSD diagnoses	8	32					1.14 (1,30)
No diagnoses in sample (RC)	4	10	−0.32 (0.15)	−2.13*			
Diagnoses in sample	8	22			−0.11 (0.11)	−1.06	
Measurement characteristics							
Quality	27	101					0.57 (2,98)
High (RC)	11	30	−0.41 (0.11)_a_	−3.57***			
Medium	14	50	−0.40 (0.11)_a_	−3.89***	−0.00 (0.09)	−0.04	
Low	13	21	−0.52 (0.12)_b_	−4.52***	−0.11 (0.12)	−0.92	

*K* = number of samples; ES = number of effect sizes; *β*_0_ = mean effects size (Cohen’s *d*); *t*_0_ = test statistic for difference mean effect with zero; *β*_1_ = regression coefficient; *t*_1_ = test statistic of difference of mean effect size with the reference category (RC); *F* (df_1_,df_2_) = test statistic for testing significance of moderator; violence/abuse includes physical and emotional abuse, neglect, sexual abuse, and violence exposure; values with the same subscripts do not differ significantly from each other at *p* < 0.05.

*p < 0.05, **p < 0.01, ***p < 0.001.

For cognitive flexibility, although the overall moderator of trauma type was not significant (*F* (2,101) = 2.62, *p* = 0.078), we found that the average effect size for single trauma exposure did not differ significantly from zero, but the mean effect sizes for violence-exposed/abused and adopted/foster care youth did, such that adopted/foster care youth performed significantly lower on cognitive flexibility than children who experienced single traumatic events, but not compared to abused youth. Results of moderator analyses for cognitive flexibility are displayed in Table 4.

### 3.4. Publication bias

We applied the PET-PEESE approach to examine publication bias in our meta-analyses. For all analyses, the PET was sufficient for assessment. The effect sizes varied significantly with the standard error for working memory (*p* < 0.001), inhibition (*p* < 0.001), and cognitive flexibility (*p* = 0.001), which makes publication selection bias likely. After assessment of the funnel plots, it seemed that there were few ‘small’ studies that reported positive effects sizes and relatively few ‘large’ studies that reported negative effect sizes. This indicates the presence of a file-drawer problem in research on trauma exposure and executive functioning in youth (Franco, Malhotra, & Simonovits, 2014).

## 4. Discussion

In the present study, we analysed the association between trauma exposure and executive functions in youth using multi-level meta-analyses. The results demonstrate small to moderate effect sizes for the association between trauma exposure and working memory (*d *= −0.49), inhibition (*d *= – 0.46), and cognitive flexibility (*d* = −0.44). These small to medium effect sizes indicate that approximately 68% of trauma-exposed youth will have a lower score on executive function tasks than youth in the control group. It is important to keep in mind, however, that we cannot draw strong conclusions about the clinical significance of the effect sizes. This is because not all outcome measures used standardized scores, and because the level of daily life impairments cannot readily be inferred from their executive functions. Executive functions work in complex ways to ultimately influence behaviour in daily life, with many factors (e.g. individual motivation, environmental support, compensatory strategies) potentially affecting this link. At the same time, because executive functions play a role in so many aspects of daily life, small to medium effect sizes can be expected to represent clinically relevant problems in trauma-exposed youth. Thus, our findings support the hypothesis that trauma exposure affects executive functions in youth.

We found that studies that used low-quality measurements showed a significantly larger effect size for the association between trauma-exposure and working memory than studies that used high-quality measurements. Researchers should be aware of the role of possible confounds when drawing conclusions based on low-quality outcome measures. Furthermore, we found that violence-exposed/abused and adopted/foster care youth demonstrated lower levels of inhibition and adopted/foster care youth showed lower levels of cognitive flexibility. Based on knowledge about early brain development and developmental trajectories of executive functions, we expected that early and prolonged exposure to traumatic events would result in problems in executive functioning compared to single trauma exposure. It is probable that adopted/foster care youth have spent these early years in an atypical, mostly emotionally unsafe environment (Merz, Harlé, Noble, & McCall, 2016), which explains why they experience more difficulties in inhibition and cognitive flexibility than single trauma-exposed youth.

Although our results suggest that trauma types influence the impact on inhibition and cognitive flexibility, we did not find that onset and duration of trauma exposure influence this relationship, and this gives us no direct indications for critical periods in the development of executive functions. This unexpected finding may be explained by the high amount of missing data (between 75% and 90%) on these moderator variables. As moderator analyses already have a lower power than the main effects analyses, this could have led to a failure to detect a meaningful difference in effect sizes across subgroups. In light of the debate about the existence of critical periods, it is interesting to note that age at testing was not a significant moderator. This goes against the widely held notion that the moderating effect of age would be stronger for younger children, as it is assumed that earlier trauma exposure has a more severe impact on cognitive function. Although at first sight perhaps counterintuitive, our findings could be explained by the fact that we did not have enough information about onset, duration, and time between cessation of trauma exposure and executive function assessment. An important suggestion for future research is, then, to clearly assess (and report) these aspects of trauma exposure to allow for further investigation of how they determine the degree of executive functioning impairments. In sum, our findings, that were based on a small amount of effect sizes should be interpreted very carefully. Based on our moderator variable for trauma type and previous neuroimaging studies, we still expect that timing and duration of trauma exposure may affect the impact of trauma exposure on executive functions (Teicher & Samson, 2016).

### 4.1. Strengths and limitations

Our study was the first meta-analysis to examine the relationship between trauma exposure and executive functions in youth with a three-level meta-analysis approach. Therefore, we could take into account the dependency among effect sizes. Our results give a systematic overview of available empirical research on this topic, and our focus on the three core executive functions (working memory, inhibition, and cognitive flexibility) added scientific and clinical value. Despite these strengths, our meta-analysis has several limitations. First, although we specifically attempted to decrease the presence of publication bias by searching for unpublished papers and dissertations, our contact attempts were mostly not answered. As our analyses indicated the presence of publication bias, our results should be interpreted carefully and ‘real’ effects may be smaller than the effects we found. Secondly, our meta-analysis was limited by missing data on theoretically important moderators such as trauma onset and duration. As there are strong indications from neuroimaging studies that the timing and duration of trauma exposure impact youth, we suggest that future research addresses these factors whenever possible. Thirdly, as both a strength and a limitation, we used various instruments that measured executive functions. This makes drawing conclusions on executive functioning in trauma-exposed youth more difficult. We handled this limitation by using a quality code on the measurement instrument, which makes us more confident about reliable outcomes. As we found that studies that used low-quality measurements showed a significantly larger effect size than studies that used high-quality measurements, future research that focuses on working memory should take this into account. As determining the quality of a task is difficult and can lead to discussion, one could, for example, combine a series of valid and reliable working memory measures in order to draw reliable conclusions instead of focusing on a sole outcome measure. Fourthly, 30–40% of studies were coded as low quality, which signals the importance for researchers to further increase the quality of their research by systematically reporting selection bias, study design, confounders, blinding, data collection methods, and withdrawal and dropouts. Fifthly, it should be noted that, as described in the introduction section, there are different types of working memory (verbal versus non-verbal) and inhibition (response inhibition and interference control). Although we aimed to investigate these differences, this was not possible because many studies used tasks that did not adequately distinguish between these different forms of working memory or inhibition. For example, many non-verbal working memory tasks do not exclude verbal working memory strategies, and there is little consensus about the categorization of Stroop-like tasks in response inhibition or interference control (e.g. Geurts, Van den Bergh, & Ruzzano, 2014). Finally, it is also important to note that we could not test causal pathways or investigate underlying neurobiological mechanisms in our meta-analysis. While exposure to trauma may impact executive functioning, it could also be that deficits in executive functions may make individuals more at risk for exposure to traumatic events (Aupperle, Melrose, Stein, & Paulus, 2012). Therefore, future research should investigate this possibility to prevent trauma exposure and, in turn, its severe consequences such as PTSD, and internalizing and externalizing problems.

### 4.2. Future research

The dissociative subtype of PTSD was recently added to DSM-5 (American Psychiatric Association, 2013). Furthermore, empirical evidence indicates a link between dissociative symptoms and executive functions (McKinnon et al., 2016; Parlar, Frewen, Oremus, Lanius, & McKinnon, 2016). The overlap between dissociation and cognitive problems such as attention and inhibition is not yet clearly established, however. This makes it highly (clinically) relevant to assess dissociative symptoms when investigating the link between trauma exposure and executive functioning. However, there were only three studies that assessed dissociative symptoms in participants and therefore we could not include this variable. As a result, we would like to point out this important limitation of existing work and therefore strongly suggest that future research addresses dissociation when investigating the link between trauma exposure and executive functioning.

In recent literature, ‘hot’ executive functions have gained increasing attention. These functions are used for motivationally or emotionally salient goal-directed behaviour (Prencipe et al., 2011; Zelazo & Carlson, 2012). Although this was beyond the scope of our meta-analysis, which focused on the three core executive functions, it would be very interesting for future studies to look at emotionally valent tasks as specifically trauma-exposed youth may suffer from chronic activation of the stress response in the brain and attention bias towards threatening stimuli (e.g. Gunnar & Quevedo, 2007; Pine et al., 2005).

The clear linkages between trauma exposure and executive functions indicate that it is pivotal for future intervention research to address executive functions as a possible moderator of intervention effects. For example, as working memory is assumed to be fully loaded in Eye Movement Desensitization and Reprocessing (EMDR), it could be that youth with lower working memory capacities may not be able to perform two tasks simultaneously and therefore would benefit less from treatment. Another possibility could be that techniques in trauma-focused cognitive behaviour therapy make an appeal to the basic capacity to inhibit emotions, thoughts, and action to regulate intrusive thoughts.

### 4.3. Conclusions

The results of our meta-analyses highlight the relationship between trauma exposure and working memory, inhibition, and cognitive flexibility in youth, especially for adopted and foster care youth. Future research on executive function in trauma-exposed youth should take into account the differential developmental pathways of executive functions and should investigate the onset and duration of trauma exposure. To draw reliable conclusions about the impact of trauma exposure in youth, researchers should use high-quality measurements. Our findings imply that clinical practice should use transdiagnostic models to incorporate problems with executive functions in their assessment and treatment guidelines for traumatized youth. Care in which trauma-exposed youth could benefit more from treatments that also focus on a broader spectrum of problems, such as executive functions, should be the next step in both research and clinical practice.

## Appendix A

**Table A1.** Search strategy for the Embase database.

1. Aircraft accident/ or destruction/ or falling/ or structure collapse/ or traffic accident/ or exp victim/ or fire/ or explosion/ or mass disaster/ or natural disaster/ or hurricane/ or tornado/ or threat/ or assault/ or battering/ or child abuse/ or family violence/ or exp partner violence/ or battered woman/ or ethnic conflict/ or genocide/ or homicide/ or human trafficking/ or infanticide/ or physical violence/ or torture/ or sexual aggression/ or exp female genital mutilation/ or sex trafficking/ or sexual coercion/ or sexual exploitation/ or exp sexual abuse/ or exp rape/ or exp sexual abuse/ or exp sexual harassment/ or exp child abuse/ or emotional abuse/ or physical abuse/ or war crime/ or war/ or kidnapping/ or abduction/ or hostage/ or stalking/ or detention/ or suicide/ or suicide attempt/ or exp child death/ or early life stress/ or orphanage/ or foster care/ or earthquake/ or incest/
2. (psychiatr* or psychol* or neurocogn* or cognit* or neuropsych* or psycho or psychosocial).ab,jx,kw,ti.
3. 1 and 2
4. 2 and (mass fatalit* or catastrophe or disaster? or accident? or aircraft crash or destruction or annihilation or falling or fall? or collapse or automobile collision or flood* or inundation or hurricane* or tornado* or cyclone* or typhoon* or twister or earthquake* or tsunami* or fire or wildfire* or blast* or threat or harassment or assault or battering or ethnic conflict or racial conflict or genocide or ethnic cleansing or ethnocide or homicide or assassination or murder or trafficking or infanticide or torture or sexual aggression or female genital mutilation or circumcised wom?n or female circumcision or female genital circumcision or female genital cutting or FGM or ritual female genital surgery or sexual coercion or sexual exploitation or forced prostitution or rape or sexual abuse or molestation or sex abuse? or frotteurism or child abuse or abused child or child negligence or neglected child or child neglect or emotional abuse or emotional neglect or physical neglect or physical abuse or battered wom?n or partner abuse or spouse abuse or wife beating or battered wife or shooting or armed attack or war or warfare or child soldier or unwanted child or abandoned child or kidnap* or abduct* or hostage or stalk* or detention or police custody or arrested or accidental death or ((suicide or self killing or suicidal) adj3 witness*) or (death adj3 (sibl* or brother or sister)) or unnatural death or death bod* or corpse? or psychotrauma or emotional trauma or mental trauma or psychical trauma or psychological trauma or psychic trauma or early life stress or orphan or orphanage or institutional care or rejected child or foster care or foster family or foster home or drowning or volcano eruption or child maltreatment or child mistreatment or killing* or wrongful death* or sex offense* or physical maltreatment or parental death or maternal death or paternal death or shell shock or corporal punishment or punishment or psychological abuse or battered females or incest* or acute stress or traumatic stress or Victim? or violent or violence or traumatic or trauma or psychotraum* or maltreatment or abuse or neglect or deprivation or bullying or bullied).ab,kw,ti.
5. posttraumatic stress disorder/ or acute stress disorder/ or exp psychotrauma/ or exp psychotrauma assessment/ or bullying/
6. (ptsd or ptss or posttraumatic stress or post traumatic stress or posttraumatic symptom? or post traumatic symptom? or bullying or bullied or cyberbullying).ab,kw,ti.
7. (life change event? and trauma*).ab,kw,sh,ti.
8. or/3–7 [traumatic events]
9. adolescent/ or child/ or minors/ or child, abandoned/ or exp child, exceptional/ or child, orphaned/ or child, unwanted/
10. (young adult? or childhood or youth* or boy? or girl? or sibling* or child or children or adolescents or adolescence or juvenile or minors or teen or teens or teenage* or young people or toddler? or pre school* or preschool* or infancy or infant? or school age).ab,kw,ti.
11. (pe?diatr* or child*).jw.
12. or/9–11 [0–25 yrs]
13. (((school or campus or universit* or bus) and (accident? or shoot* or massacre or violence or disaster?)) or utoya).ab,kw,ti.
14. 8 and (12 or 13)
15. *executive function/ or exp *attention/ or exp *memory/ or *problem solving/ or *self control/ or *self evaluation/ or *creativity/ or *delay discounting/ or *attentional bias/ or *memory bias/ or exp *‘inhibition (psychology)’/
16. (executive function? or executive dysfunction? or dysexecutive syndrome or executive control or cognitive control or (inhibitory adj2 control) or self-control or selective attention or cognitive inhibition or interference control or focused attention or attentional inhibition or attentional control or endogenous attention or voluntary attention or top-down attention or active attention or goal driven attention or executive attention or delaying gratification or delayed gratification or Temporal Discounting or Intertemporal Preference* or Intertemporal Decision Making or Deferred Gratification or response inhibition or working memory or verbal working memory or nonverbal working memory or visual spatial working memory or cognitive flexibility or cognitive development or set shifting or mental flexibility or mental set shifting or creativity or verbal fluency or category fluency or semantic fluency or task switching or planning or reasoning or problem-solving or fluid intelligence or self regulation or effortful control).ab,kw,ti.
17. or/15–16
18. 14 and 17
19. exp executive function test/
20. (Conners Continuous Performance TEST or (Stroop adj3 (task? or Test)) or D-KEFS or Delis-Kaplan Executive Function System or Wisconsin Card Sorting Test or WCST or card sorting test or Porteus maze? or Rey-Osterrieth Complex Figure or RCFT or (brief adj3 (behavior or task? or test* or inventory)) or ‘behavior rating inventory of executive functions’ or BADS or ‘behavioural assessment of the dysexecutive syndrome’ or ‘Stop/go’ or ‘stop/signal’ or ‘Go/no go’ or Flanker or Dimensional card sorting task or Self-ordered pointing task or Conflict task or Gambling task or attention bias).ab,kw,ti. [specific tests]
21. 19 or 20
22. 14 and 21
23. Bender Gestalt Test/ or ‘Kaufman assessment battery for children’/ or ‘test of everyday attention’/ or Wechsler adult intelligence scale/ or Wechsler intelligence scale for children/ or Wechsler memory scale/ or exp maze test/
24. (NEPSY or neuropsychological assessment or KABC or kaufman assessment or ‘WJ-III’ or woodcock johnson or ‘Test of Everyday Attention’ or WISC or wechsler intelligence or WRAML2 or ‘wide range of assessment and learning’ or ‘Test of Problem Solving’ or differential ability scales or VMI or Visual Motor Integration or cognitive Assessment System or children memory scale or Cambridge Neuropsychological Test Automated Battery or CANTAB).ab,kw,ti. [generic relevant tests]
25. 23 or 24
26. 14 and 25
27. or/18,22,26
28. (tbi or traumatic brain or abi or acquired brain).kw,sh,ti.
29. 27 not 28
30. (animal/ or animal experiment/ or animal model/ or nonhuman/ or rat/ or mouse/ or (rat or rats or mouse or mice).ti.) not human/
31. 29 not 30
32. remove duplicates from 31

**Table A2.** Search strategy for the MEDLINE database.

1. Accidental falls/ or accidents, Aviation/ or Accidents, home/ or accidents, traffic/ or drowning/ or mass casualty incidents/ or disaster victims/ or explosions/ or cyclonic storms/ or earthquakes/ or tornadoes/ or exp ethnic violence/ or exp child abuse/ or physical abuse/ or exp intimate partner violence/ or domestic violence/ or spouse abuse/ or torture/ or battered woman/ or exp genocide/ or homicide/ or exp sex offenses/ or infanticide/ or sexual harassment/ or circumcision, female/ or exp war crimes/ or stalking/ or parental death/ or maternal death/ or suicide, attempted/ or suicide, assisted/ or foster home care/ or orphanages/ or incest/
2. (psychiatr* or psychol* or neurocogn* or cognit* or neuropsych* or psycho or psychosocial).ab,jw,kf,ti.
3. 1 and 2
4. 2 and (mass fatalit* or catastrophe or disaster? or accident? or aircraft crash or destruction or annihilation or falling or fall? or collapse or automobile collision or flood* or inundation or hurricane* or tornado* or cyclone* or typhoon* or twister or earthquake* or tsunami* or fire or wildfire* or blast* or threat or harassment or assault or battering or ethnic conflict or racial conflict or genocide or ethnic cleansing or ethnocide or homicide or assassination or murder or trafficking or infanticide or torture or sexual aggression or female genital mutilation or circumcised wom?n or female circumcision or female genital circumcision or female genital cutting or FGM or ritual female genital surgery or sexual coercion or sexual exploitation or forced prostitution or rape or sexual abuse or molestation or sex abuse? or frotteurism or child abuse or abused child or child negligence or neglected child or child neglect or emotional abuse or emotional neglect or physical neglect or physical abuse or battered wom?n or partner abuse or spouse abuse or wife beating or battered wife or shooting or armed attack or war or warfare or child soldier or unwanted child or abandoned child or kidnap* or abduct* or hostage or stalk* or detention or police custody or arrested or accidental death or ((suicide or self killing or suicidal) adj3 witness*) or (death adj3 (sibl* or brother or sister)) or unnatural death or death bod* or corpse? or psychotrauma or emotional trauma or mental trauma or psychical trauma or psychological trauma or psychic trauma or early life stress or orphan or orphanage or institutional care or rejected child or foster care or foster family or foster home or drowning or volcano eruption or child maltreatment or child mistreatment or killing* or wrongful death* or sex offense* or physical maltreatment or parental death or maternal death or paternal death or shell shock or corporal punishment or punishment or psychological abuse or battered females or incest* or acute stress or traumatic stress or Victim? or violent or violence or traumatic or trauma or psychotraum* or maltreatment or abuse or neglect or deprivation or bullying or bullied).ab,kf,ti.
5. exp ‘Trauma and Stressor Related Disorders’/ or bullying/
6. (ptsd or ptss or posttraumatic stress or post traumatic stress or posttraumatic symptom? or post traumatic symptom? or bullying or bullied or cyberbullying).ab,kf,ti.
7. (life change event? and trauma*).ab,kf,sh,ti.
8. or/3–7 [traumatic events]
9. adolescent/ or child/ or minors/ or child, abandoned/ or exp child, exceptional/ or child, orphaned/ or child, unwanted/
10. (young adult? or childhood or youth* or boy? or girl? or sibling* or child or children or adolescents or adolescence or juvenile or minors or teen or teens or teenage* or young people or toddler? or pre school* or preschool* or infancy or infant? or school age).ab,kf,ti.
11. (pe?diatr* or child*).jw.
12. or/9–11 [0–25 yrs]
13. (((school or campus or universit* or bus) and (accident? or shoot* or massacre or violence or disaster?)) or utoya).ab,kf,ti.
14. 8 and (12 or 13)
15. executive function/ or attention/ or Memory, Short-Term/ or exp problem solving/ or self control/ or creativity/ or delay discounting/ or ‘Inhibition (Psychology)’/
16. (executive function? or executive dysfunction? or dysexecutive syndrome or executive control or cognitive control or (inhibitory adj2 control) or self-control or selective attention or cognitive inhibition or interference control or focused attention or attentional inhibition or attentional control or endogenous attention or voluntary attention or top-down attention or active attention or goal driven attention or executive attention or delaying gratification or delayed gratification or Temporal Discounting or Intertemporal Preference* or Intertemporal Decision Making or Deferred Gratification or response inhibition or working memory or verbal working memory or nonverbal working memory or visual spatial working memory or cognitive flexibility or cognitive development or set shifting or mental flexibility or mental set shifting or creativity or verbal fluency or category fluency or semantic fluency or task switching or planning or reasoning or problem-solving or fluid intelligence or self regulation or effortful control).ab,kf,ti.
17. or/15–16
18. 14 and 17
19. (Conners Continuous Performance TEST or (Stroop adj3 (task? or Test)) or D-KEFS or Delis-Kaplan Executive Function System or Wisconsin Card Sorting Test or WCST or card sorting test or Porteus maze? or Rey-Osterrieth Complex Figure or RCFT or (brief adj3 (behavior or task? or test* or inventory)) or ‘behavior rating inventory of executive functions’ or ‘BEHAVIOURAL ASSESSMENT OF THE DYSEXECUTIVE SYNDROME’ or ‘Stop/go’ or ‘stop/signal’ or ‘Go/no go’ or Flanker or Dimensional card sorting task or Self-ordered pointing task or Conflict task or Gambling task or attention bias).ab,kf,ti. [specific tests]
20. 14 and 19
21. Wechsler Scales/
22. (NEPSY or neuropsychological assessment or KABC or kaufman assessment or ‘WJ-III’ or woodcock johnson or ‘Test of Everyday Attention’ or WISC or wechsler intelligence or WRAML2 or ‘wide range of assessment and learning’ or ‘Test of Problem Solving’ or differential ability scales or VMI or Visual Motor Integration or cognitive Assessment System or children memory scale or Cambridge Neuropsychological Test Automated Battery or CANTAB).ab,kf,ti.
23. 21 or 22 [generic relevant tests]
24. 14 and 23
25. or/18,20,24
26. (tbi or traumatic brain or abi or acquired brain).kf,sh,ti.
27. 25 not 26
28. animals/ not humans/
29. 27 not 28
30. remove duplicates from 29
31. limit 30 to (dutch or english)

**Table A3.** Search strategy for the PsycINFO database.

1. Falls/ or home accidents/ or pedestrian accidents/ or exp transportation accidents/ or exp disasters/ or threat/ or coercion/ or punishment/ or school violence/ or physical abuse/ or emotional abuse/ or exp harassment/ or victimization/ or human trafficking/ or kidnapping/ or battered females/ or domestic violence/ or exposure to violence/ or exp partner abuse/ or exp sex offenses/ or circumcision/ or battered females/ or kidnapping/ or exp suicide/ or homicide/ or emotional trauma/ or foster children/ or foster care/ or orphans/ or orphanages/
2. (psychiatr* or psychol* or neurocogn* or cognit* or neuropsych* or psycho or psychosocial).ab,jx,id,ti.
3. 1 and 2
4. 2 and (mass fatalit* or catastrophe or disaster? or accident? or aircraft crash or destruction or annihilation or falling or fall? or collapse or automobile collision or flood* or inundation or hurricane* or tornado* or cyclone* or typhoon* or twister or earthquake* or tsunami* or fire or wildfire* or blast* or threat or harassment or assault or battering or ethnic conflict or racial conflict or genocide or ethnic cleansing or ethnocide or homicide or assassination or murder or trafficking or infanticide or torture or sexual aggression or female genital mutilation or circumcised wom?n or female circumcision or female genital circumcision or female genital cutting or FGM or ritual female genital surgery or sexual coercion or sexual exploitation or forced prostitution or rape or sexual abuse or molestation or sex abuse? or frotteurism or child abuse or abused child or child negligence or neglected child or child neglect or emotional abuse or emotional neglect or physical neglect or physical abuse or battered wom?n or partner abuse or spouse abuse or wife beating or battered wife or shooting or armed attack or war or warfare or child soldier or unwanted child or abandoned child or kidnap* or abduct* or hostage or stalk* or detention or police custody or arrested or accidental death or ((suicide or self killing or suicidal) adj3 witness*) or (death adj3 (sibl* or brother or sister)) or unnatural death or death bod* or corpse? or psychotrauma or emotional trauma or mental trauma or psychical trauma or psychological trauma or psychic trauma or early life stress or orphan or orphanage or institutional care or rejected child or foster care or foster family or foster home or drowning or volcano eruption or child maltreatment or child mistreatment or killing* or wrongful death* or sex offense* or physical maltreatment or parental death or maternal death or paternal death or shell shock or corporal punishment or punishment or psychological abuse or battered females or incest* or acute stress or traumatic stress or Victim? or violent or violence or traumatic or trauma or psychotraum* or maltreatment or abuse or neglect or deprivation or bullying or bullied).ab,id,ti.
5. posttraumatic stress disorder/ or acute stress disorder/ or exp bullying/
6. (ptsd or ptss or posttraumatic stress or post traumatic stress or posttraumatic symptom? or post traumatic symptom? or bullying or bullied or cyberbullying).ab,id,ti.
7. (life change event? and trauma*).ab,id,sh,ti.
8. or/3–7 [traumatic events]
9. (‘140’ or ‘180’ or ‘200’ or ‘320’).ag.
10. (young adult? or childhood or youth* or boy? or girl? or sibling* or child or children or adolescents or adolescence or juvenile or minors or teen or teens or teenage* or young people or toddler? or pre school* or preschool* or infancy or infant? or school age).ab,id,ti.
11. (pe?diatr* or child*).jx.
12. or/9–11 [0–25 yrs]
13. (((school or campus or universit* or bus) and (accident? or shoot* or massacre or violence or disaster?)) or utoya).ab,id,ti.
14. 8 and (12 or 13)
15. executive function/ or attention/ or exp memory/ or exp problem solving/ or self control/ or creativity/ or delay discounting/ or dysexecutive syndrome/
16. (executive function? or executive dysfunction? or dysexecutive syndrome or executive control or cognitive control or (inhibitory adj2 control) or self-control or selective attention or cognitive inhibition or interference control or focused attention or attentional inhibition or attentional control or endogenous attention or voluntary attention or top-down attention or active attention or goal driven attention or executive attention or delaying gratification or delayed gratification or Temporal Discounting or Intertemporal Preference* or Intertemporal Decision Making or Deferred Gratification or response inhibition or working memory or verbal working memory or nonverbal working memory or visual spatial working memory or cognitive flexibility or cognitive development or set shifting or mental flexibility or mental set shifting or creativity or verbal fluency or category fluency or semantic fluency or task switching or planning or reasoning or problem-solving or fluid intelligence or self regulation or effortful control).ab,id,ti.
17. or/15–16
18. 14 and 17
19. Stroop effect/ or Stroop Color Word Test/
20. (Conners Continuous Performance TEST or (Stroop adj3 (task? or Test)) or D-KEFS or Delis-Kaplan Executive Function System or Wisconsin Card Sorting Test or WCST or card sorting test or Porteus maze? or Rey-Osterrieth Complex Figure or RCFT or (brief adj3 (behavior or task? or test* or inventory)) or ‘behavior rating inventory of executive functions’ or BADS or ‘BEHAVIOURAL ASSESSMENT OF THE DYSEXECUTIVE SYNDROME’ or ‘Stop/go’ or ‘stop/signal’ or ‘Go/no go’ or Flanker or Dimensional card sorting task or Self-ordered pointing task or Conflict task or Gambling task or attention bias).ab,id,ti. [specific tests]
21. 19 or 20
22. 14 and 21
23. Bender Gestalt Test/ or Wechsler Intelligence Scale for Children/ or Woodcock Johnson Psychoeducational Battery/ or Digit span testing/ or Porteus Maze Test/ or ‘Kaufman Assessment Battery for Children’/ or Wechsler Adult Intelligence Scale/ or Wechsler Preschool Primary Scale/ or Kohs Block Design Test/
24. (NEPSY or neuropsychological assessment or KABC or kaufman assessment or ‘WJ-III’ or woodcock johnson or ‘Test of Everyday Attention’ or WISC or wechsler intelligence or WRAML2 or ‘wide range of assessment and learning’ or ‘Test of Problem Solving’ or differential ability scales or VMI or Visual Motor Integration or cognitive Assessment System or children memory scale or Cambridge Neuropsychological Test Automated Battery or CANTAB).ab,id,ti. [generic relevant tests]
25. 23 or 24
26. 14 and 25
27. or/18,22,26
28. (tbi or traumatic brain or abi or acquired brain).id,sh,ti.
29. 27 not 28
30. cognitive control.ab,id,ti.
31. (executive function? or executive dysfunction? or dysexecutive syndrome or executive control or cognitive control or (inhibitory adj2 control)).ab,id,ti.
32. (self-control or selective attention or cognitive inhibition or interference control or focused attention or attentional inhibition or attentional control).ab,id,ti.
33. (task switching or planning or reasoning or problem-solving or fluid intelligence or self regulation or effortful control).ab,id,ti.
34. (cognitive development or set shifting or mental flexibility or mental set shifting or creativity or verbal fluency or category fluency or semantic fluency).ab,id,ti.
35. (response inhibition or working memory or verbal working memory or nonverbal working memory or visual spatial working memory or cognitive flexibility).ab,id,ti.
36. (gratification or delayed gratification or Temporal Discounting or Intertemporal Preference* or Intertemporal Decision Making or Deferred Gratification).ab,id,ti.
37. (endogenous attention or voluntary attention or top-down attention or active attention or goal driven attention or executive attention or delaying gratification).ab,id,ti.
38. 8 and 9
39. 13 or 38
40. 39 and (17 or 21 or 25)
41. 40 not 28
42. limit 41 to (human and (dutch or english))
43. limit 42 to (‘0100 journal’ or ‘0110 peer-reviewed journal’ or ‘0400 dissertation abstract’)

## Appendix B

**Table B1.** Eligibility assessment criteria.

*Types of studies*
We included studies that compared trauma-exposed youth with a control group in terms of inhibition, working memory, or cognitive flexibility
We included studies that investigated the association between trauma exposure and inhibition, working memory, or cognitive flexibility in youth, with the exception of samples of orphans, institutionalized and adopted youth
We included studies that compared orphans, institutionalized, and adopted youth with a control group in terms of inhibition, working memory, or cognitive flexibility
*Types of participants*
We included samples with traumatized youth aged 0–25 years, in which the upper age limit could not exceed 25 years of age
We excluded samples when participants were reported to have physical disabilities or illness: such as traumatic brain injury, poisoning, cancer, heart problems, epilepsy
*Trauma criteria*
Population: orphans, foster children, adopted children
Experiencing/witnessing/hearing about:
Natural disaster (e.g. hurricane, earthquake) Fire/explosion Accident (traffic, school, home, neighbourhood) Bullying (extreme) Physical attack (beaten, kicked, etcetera) Shooting War/community violence Verbal abuse Domestic violence Rape, sexual abuse Stalking Police arrest Physical neglect Emotional neglect Abduction/kidnapping Severe illness Death by violence Death of a loved one
*Outcome measures*
Working memory: Visuospatial working memory Spatial working memory Verbal working memory
Inhibition: Response inhibition Inhibitory control Interference control Cognitive inhibition Selective attention Focused attention Effortful control
Cognitive flexibility: Set shifting Task switching Shifting
*Correlations or means*
We included studies that reported raw correlations between measures or means and standard deviations between two groups

## Appendix C

**Table C1.** Coding scheme.

Variable	Variable labels
Study characteristics	
PaperID	Paper identification number (001, 002, 003, etc.)
SampleID	Sample identification number (001, 002, 003, etc.)
ESID	Effect size identification number (001, 002, 003, etc.)
Authors	Author names
Year	Publication year
Publication status	1 = published, 0 = not published
N	Number of participants in total sample
AgeMean	Mean age, total sample
AgeSD	Standard deviation age, total sample
Gender	Percentage girls in total sample
Ethnicity	Percentage minority ethnicity in total sample
SES	1 = controlled for SES, 0 = not controlled for SES
N_control	Number of participants in control group
AgeMean_control	Mean age, control group
AgeSD_control	Standard deviation age, control group
Gender_control	Percentage girls in control group
Ethnicity_control	Percentage minority ethnicity in control group
N_trauma	Number of participants in trauma group
AgeMean_trauma	Mean age, trauma group
AgeSD_trauma	Standard deviation age, trauma group
Gender_trauma	Percentage girls in trauma group
Ethnicity_trauma	Percentage minority ethnicity in trauma group
Trauma characteristics	
PTSD_measure	PTSD measurement instrument: 1 = CRIES (child), 2 = CRIES (parent), 3 = TSCC, 4 = PDS, 5 = UCLA PTSD index, 6 = CAPS-CA, 7 = PCL, 8 = PCL-C, 9 = TSCYC, 10 = PSSC, 11 = KSADS, 12 = observation, 13 = mini-KID, 14 = IES, 15 = psychiatric evaluation, 16 = SCDID
PTSD_Diagnosis	1 = PTSD diagnoses in sample, 0 = no PTSD diagnoses in sample
Type_trauma	1 = disaster, 2 = fire or explosion, 3 = vehicle accident, 4 = accident, 5 = overall abuse, 6 = overall neglect, 7 = physical abuse/threat, 8 = verbal abuse/threat, 9 = emotional neglect, 10 = physical neglect, 11 = domestic violence, 12 = sexual abuse/rape, 13 = (witness) shooting, 14 = stalking, 15 = person in family arrested, 16 = severe bullying (with physical threat), 17 = abduction, 18 = witness of a violent death, 19 = death of a loved one, 20 = adoption/foster care with known history of abuse or neglect, 21 = adoption/foster care with unknown history, 22 = severe illness or medical condition in loved one, 23 = indirect victimization 24 = community violence (later subsumed into 1 = single trauma exposure, 2 = violence exposed/abused/neglect, 3 = adopted or foster care youth)
Onset	Mean age (years) of onset of trauma exposure
Duration	Mean age (years) of duration of trauma exposure
Measurement characteristics	
WM_Task	Working memory outcome measure
WM_mean_control	Mean score on working memory outcome measure for control group
WM_SD_control	Standard deviation on working memory outcome measure for control group
WM_mean_trauma	Mean score on working memory outcome measure for trauma group
WM_SD_trauma	Standard deviation on working memory outcome measure for trauma group
WM_correlation	Correlation between trauma exposure and working memory outcome measure
WM_quality	1 = high, 2 = medium, 3 = low
INH_Task	Inhibition outcome measure
INH_mean_control	Mean score on inhibition outcome measure for control group
INH_SD_control	Standard deviation on inhibition outcome measure for control group
INH_mean_trauma	Mean score on inhibition outcome measure for trauma group
INH_SD_trauma	Standard deviation on inhibition outcome measure for trauma group
INH_correlation	Correlation between trauma exposure and inhibition outcome measure
INH_quality	1 = high, 2 = medium, 3 = low
FLEX_Task	Cognitive flexibility outcome measure
FLEX_mean_control	Mean score on cognitive flexibility outcome measure for control group
FLEX_SD_control	Standard deviation on cognitive flexibility outcome measure for control group
FLEX_mean_trauma	Mean score on cognitive flexibility outcome measure for trauma group
FLEX_SD_trauma	Standard deviation on cognitive flexibility outcome measure for trauma group
FLEX_correlation	Correlation between trauma exposure and cognitive flexibility outcome measure
FLEX_quality	1 = high, 2 = medium, 3 = low

## Appendix D

**Table D1.** Quality coding of included working memory outcome measures.

Task – outcome measure	Measures	Quality
*(WISC) Digit Span*		
Overall	Verbal working memory	Low
Backwards–forwards	Verbal working memory	High
Backwards	Verbal working memory	Medium
*WISC WMI index*	Working memory	Low
*CANTAB SWM*		
SWM between errors 4–8 boxes	Spatial working memory	Medium
Within errors 4–8 boxes	Spatial working memory	Medium
Double errors	Spatial working memory	Medium
Total errors 4–8 boxes (key outcome)	Spatial working memory	High
Strategy (key outcome)	Spatial working memory	High
Mean score	Spatial working memory	Low
*CANTAB Spatial Span (SSP)*		
SSP errors	Spatial working memory	High
SSP length	Spatial working memory	High
SSP strategy	Spatial working memory	High
SSP latency	Spatial working memory	Medium
*WJ-II*		
Numbers reversed	Verbal working memory	Medium
*NEUROPSI*		
Digit backwards span	Verbal working memory	Medium
Spatial backwards span	Spatial working memory	Medium
*CAT*		
Spatial working memory (overall)	Spatial working memory	Low
*Combined tasks*		
Digit Span (WISC) + Corsi Block test	Working memory (spatial + verbal)	Medium
*Listening recall task*	Verbal working memory	Medium
*Odd-one-out*	Verbal working memory	Medium
*Spin the pots (# stickers)*	Working memory (spatial)	Medium
*Six boxes (scrambled)*	Working memory (spatial)	Medium
*BRIEF*		
Working memory subscale	Working memory	Low

**Table D2.** Quality coding of included inhibition outcome measures.

Task – outcome measure	Measures	Quality
*Stroop*		
Errors card III	Interference control	Medium
RT card III	Interference control	Medium
Interference score (card III – II)	Interference control	High
*Delis Kaplan Color Word Interference*		
Mean score	Interference control	Medium
Errors card III	Interference control	Medium
Contrast time/errors (difference card III–II/I)	Interference control	High
*Go/no-go*		
Percentage correct no-go responses	Response inhibition	High
Percentage errors of commission	Response inhibition	High
Reaction time errors of commission	Response inhibition	High
Total percentage correct	Response inhibition	Low
Total reaction time	Response inhibition	Low
*Conners Performance Test II*		
Commission errors	Response inhibition	High
*Stop Signal Test*		
SSRT	Inhibit prepotent response	High
Proportion successful stops	Inhibit prepotent response	Medium
Stop signal delay	Inhibit prepotent response	Medium
Mean probability of inhibition over all delay intervals corrected for omission errors	Inhibit prepotent response	High
*Flanker*		
Accuracy incongruent	Interference control	Medium
RT incongruent	Interference control	Medium
Incongruent–congruent RT	Interference control	High
Interference score	Interference control	High
*Nepsy*		
Knock and tap: accuracy score	Motor inhibition	High
Statue: accuracy score	Motor inhibition	High
*Gradual Onset Continuous Performance Task*		
Slope of commission errors	Interference control	High
*Logan Stop-Change*		
% Correct responses for tone delay trials	Interference control	Medium
Mean reaction time for tone delay trials	Interference control	High
*Change task (McClure)*		
CSRT	Interference control	High
*Three pegs task*	Prepotent response inhibition	Medium
*Tapping task*	Prepotent response inhibition	Medium
*Day night*		
Proportion correct test trials	Interference control	Low
*NEUROPSI*		
Motor functions (Go/no-go + Luria’s)	Inhibition	Low
*BRIEF*		
Inhibition subscale	Inhibition	Low
*Verbal Inhibition/Motor Inhibition Task*		
Combined number of errors	Inhibition	low
*Luria’s hand game based task*		
Combined number of errors	Inhibition	Low

Assignment of quality is partly based on the paper of Geurts et al. (2014).

**Table D3.** Quality coding of included cognitive flexibility outcome measures.

Task – outcome measure	Measures	Quality
*Trail Making Test (TMT)*		
TMT-B	Cognitive flexibility	Medium
TMT-A + B	Cognitive flexibility	Low
TMT-B **– **A	Cognitive flexibility	High
*DKEFS Category switching*		
Average score CF **–** average score switching	Verbal flexibility	High
Average score Con1 + 2 – raw score Con3	Non-verbal flexibility	High
*DCCS*		
Highest level achieved	Set shifting	Low
*CANTAB IED*		
Total errors/errors block 6/errors block 8	Set shifting	Medium
Total errors adjusted	Set shifting	Medium
Stages completed	Set shifting	Medium
EDS errors	Set shifting	High
PRE ED errors	Set shifting	Medium
Total trials	Set shifting	Medium
Total trials adjusted	Set shifting	Medium
Mean score	Set shifting	Low
WCST		
Perseverative errors	Set shifting	High
Perseverative responses	Set shifting	Medium
Total errors	Set shifting	Low
Categories completed	Set shifting	Low
Failure to maintain set	Set shifting	High
*Flexible item task*		
Proportion correct	Set shifting	Medium
*BRIEF*		
Cognitive flexibility subscale	Cognitive flexibility	Low
*Cognitive Flexibility Inventory*	Cognitive flexibility	Low
Combined tasks		
TMT-B + WCST perseveration	Cognitive flexibility	Medium

Assignment of quality is partly based on the paper of Geurts, Corbett, and Solomon (2009).

**Table D4.** Excluded tasks and outcome measures.

Tasks – outcome measures	Measures
*Go/no-go*	
Correct Go responses (number of correct ‘go’ responses)	Selective attention
% Correct Go responses (percentage of ‘go’ trials correct)	Selective attention
Incorrect Go responses (number of incorrect ‘go’ responses)	Selective attention
Go trial non-responses (non-responses on ‘go’ trials)	Selective attention
Mean Go trial RT (mean reaction time of correct ‘go’ responses)	Selective attention
*Conner’s Performance Test II*	
Correct detection	Selective attention
RT	Selective attention
Omission errors	Selective attention
Variability	Sustained attention
*Stop Signal Test*	
SSD	Time interval between go and stop signals
Go RT	Selective attention
Direction errors	Selective attention
*Flanker*	
Accuracy congruent	Selective attention
RT congruent	Selective attention
Combined scores congruent	Selective attention
*Logan Stop-Change*	
Go RT	Selective attention
SSD	Selective attention
*WISC Digit Span*	
Forwards	Attention/short-term memory
*CANTAB SWM*	
Mean time to first response	Speed
Sentence repetition	Short-term memory
*TMT*	
TMT-A	Psychomotor speed
*Digital Vigilance Test*	Vigilance + alertness
*COWAT*	
Animal naming	Verbal fluency
Total words	Verbal fluency
*Grooved Pegboard*	Planning + psychomotor speed
*California Verbal Learning Test*	
List A	Verbal learning
List B	Verbal learning
Short delay free recall	Verbal learning
Long delay free recall	Verbal learning
Discriminability	Verbal learning
*WISC-III*	
Block design	Visual–motor coordination
Object assembly	Visual organizing/reasoning
Coding	Visual short-term memories
Similarities	Reasoning
Arithmetic	Arithmetic abilities
*Rey Osterrieth Complex Figure recall*	Memory
*Rey Osterrieth Complex Figure copy*	Visual–spatial ability
*Money Road Map*	Left–right discrimination
*Judgement of Line Orientation*	Visual–spatial ability
*Tower of London*	Planning
*Reading span of Daneman & Carpenter*	Short-term memory
*Self-control scale*	Self-control
*Conflict task (Egner 2008)*	Conflict interference (emotional)
*Hayling sentence repetition*	Selective attention
*Sentence repetition span*	Verbal memory:
*Paired Associate Learning test CANTAB*	Visual memory + new learning
*Dot-probe*	Attention bias
*Retrospective Self Report of Inhibition (RSRI)*	Behavioural disinhibition (trait)
*Stanford Binet Sentence, Objects and Digits*	Short-term memory
*Bayley scales*	Overall cognitive function
*BIS/BAS scales*	Behavioural inhibition (trait)
*Barrat Impulsivity Scale*	Impulsivity
*Children’s Behavior Questionnaire (CBQ)*	Temperament
*FDI index WISC*	Attention
*Gift delay task*	Behavioural inhibition
*Composite score of DCCS, Day/Night/CBCL*	Overall (executive) functioning
*WCST*	
Non-perseverative errors	Random errors

## Appendix E

**References of papers used in meta-analyses for working memory, inhibition, and cognitive flexibility**

Almas, A. N., Degnan, K. A., Nelson, C. A., Zeanah, C. H., & Fox, N. A. (2016). IQ at age 12 following a history of institutional care: Findings from the Bucharest Early Intervention Project. *Developmental Psychology*, *52*(11), 1858–1866. doi:10.1037/dev0000167

Augusti, E.-M., & Melinder, A. (2013). Maltreatment is associated with specific impairments in executive functions: A pilot study. *Journal of Traumatic Stress, 26*, 780–783. doi:10.1002/jts

Barrera, M., Calderon, L., Bell, V., Calderón, L., & Bell, V. (2013). The cognitive impact of sexual abuse and PTSD in children: A neuropsychological study. *Journal of Child Sexual Abuse, 22*(6), 625–638. doi:10.1080/10538712.2013.811141

Bauer, P. M., Hanson, J. L., Pierson, R. K., Davidson, R. J., & Pollak, S. D. (2009). Cerebellar volume and cognitive functioning in children who experienced early deprivation. *Biological Psychiatry, 66*(12), 1100–1106. doi:10.1016/j.biopsych.2009.06.014

Beers, S. R., & De Bellis, M. D. (2002). Neuropsychological function in children with maltreatment-related posttraumatic stress disorder. *The American Journal of Psychiatry, 159*, 483–486. doi:10.1176/appi.ajp.159.3.483

Bos, K. J., Fox, N., Zeanah, C. H., & Nelson III, C. A. (2009). Effects of early psychosocial deprivation on the development of memory and executive function. *Frontiers in Behavioral Neuroscience, 3*, 1–16. doi:10.3389/neuro.08.016.2009

Brett, Z. H., Sheridan, M., Humphreys, K., Smyke, A., Gleason, M. M., Fox, N., … Drury, S. (2014). A neurogenetics approach to defining differential susceptibility to institutional care. *International Journal of Behavioral Development*, 1–11. doi:10.1177/0165025414538557

Bruce, J., McDermott, J. M., Fisher, P. A., & Fox, N. A. (2009). Using behavioral and electrophysiological measures to assess the effects of a preventive intervention: A preliminary study with preschool-aged foster children. *Prevention Science, 10*(2), 129–140. doi:10.1007/s11121-008-0115-8

Bücker, J., Kapczinski, F., Post, R., Cereser, K. M., Szobot, C., Yatham, L. N., … Kauer-Sant’Anna, M. (2012). Cognitive impairment in school-aged children with early trauma. *Comprehensive Psychiatry, 53*, 758–764. doi:10.1016/j.comppsych.2011.12.006

Burgers, D. E., & Drabick, D. A. G. (2016). Community violence exposure and generalized anxiety symptoms: Does executive functioning serve a moderating role among low income, urban youth? *Journal of Abnormal Child Psychology, 44*, 1543–1557. doi:10.1007/s10802-016-0144-x

Cardona, J. F., Manes, F., Escobar, J., López, J., & Ibáez, A. (2012). Potential consequences of abandonment in preschool-age: Neuropsychological findings in institutionalized children. *Behavioural Neurology, 25*, 291–301. doi:10.3233/BEN-2012-110205

Carrion, V. G., Garrett, A., Menon, V., Weems, C. F., & Reiss, A. L. (2008). Posttraumatic stress symptoms and brain function during a response-inhibition task: An fMRI study in youth. *Depression and Anxiety, 25*, 514–526. doi:10.1002/da.20346

Cecil, C. A. M., Viding, E., McCrory, E. J., & Gregory, A. M. (2015). Distinct mechanisms underlie associations between forms of childhood maltreatment and disruptive nocturnal behaviors. *Developmental Neuropsychology, 40*(3), 181–199. doi:10.1080/87565641.2014.983636

Cowell, R. A., Cicchetti, D., Rogosch, F. A., & Toth, S. L. (2015). Childhood maltreatment and its effect on neurocognitive functioning: Timing and chronicity matter. *Development and Psychopathology, 27*, 521–533. doi:10.1017/S0954579415000139

De Bellis, M. D., Woolley, D. P., & Hooper, S. R. (2013). Neuropsychological findings in pediatric maltreatment. *Child Maltreatment, 18*(3), 171–183. doi:10.1177/1077559513497420

Dileo, J. F., Brewer, W., Northam, E., Yucel, M., Anderson, V., Brewer, W., … Anderson, V. (2017). Investigating the neurodevelopmental mediators of aggression in children with a history of child maltreatment: An exploratory field study. *Child Neuropsychology, 23*(6), 655–677. doi:10.1080/09297049.2016.1186159

Eigsti, I.-M., Weitzman, C., Schuh, J., de Marchena, A., & Casey, B. J. (2011). Language and cognitive outcomes in internationally adopted children. *Development and Psychopathology, 23*, 629–646. doi:10.1017/S0954579411000204

Fishbein, D., Warner, T., Krebs, C., Trevarthen, N., Falnnery, B., & Hammond, J. (2009). Differential relationships between personal and community stressors and children’s neurocognitive functioning. *Child Maltreatment, 14*(4), 299–315. doi:10.1177/1077559508326355

Fox, N. A., Almas, A. N., Degnan, K. A., Nelson, C. A., & Zeanah, C. H. (2011). The effects of severe psychosocial deprivation and foster care intervention on cognitive development at 8 years of age: Findings from the Bucharest Early Intervention Project. *Journal of Child Psychology and Psychiatry and Allied Disciplines*, 52(9), 919–928. doi: 10.1111/j.1469-7610.2010.02355.x

Fu, F., & Chow, A. (2017). Traumatic exposure and psychological well-being: The moderating role of cognitive flexibility. *Journal of Loss and Trauma, 22*, 24–35. doi:10.1080/15325024.2016.1161428

Gustafsson, H. C., Coffman, J. L., & Cox, M. J. (2015). Intimate partner violence, maternal sensitive parenting behaviors, and children’s executive functioning. *Psychology of Violence*, *5*(3), 266–274. doi:10.1037/a0037971

Gustafsson, H. C., Coffman, J. L., Harris, L. S., Langley, H. A., Ornstein, P. A., & Cox, M. J. (2013). Intimate partner violence and children’s memory. *Journal of Family Psychology, 27*, 937–944. doi:10.1037/a0034592

Hanson, J. L., Chung, M. K., Avants, B. B., Rudolph, K. D., Shirtcliff, E. A., Gee, J. C., … Pollak, S. D. (2012). Structural variations in prefrontal cortex mediate the relationship between early childhood stress and spatial working memory. *Journal of Neuroscience*, 32, 7917–7925. Doi: 10.1523/JNEUROSCI.0307-12.2012.

Helder, E. J., Behen, M. E., Wilson, B., Muzik, O., & Chugani, H. T. (2014). Language difficulties in children adopted internationally: Neuropsychological and functional neural correlates. *Child Neuropsychology, 20*(4), 470–492. doi:10.1080/09297049.2013.819846

Hostinar, C. E., Stellern, S. A., Schaefer, C., Carlson, S. M., & Gunnar, M. R. (2012). Associations between early life adversity and executive function in children adopted internationally from orphanages. *Proceedings of the National Academy of Sciences of the United States of America, 109*, 17208–17212. doi:10.1073/pnas.1121246109

Kavanaugh, B., & Holler, K. (2014). Executive, emotional, and language functioning following childhood maltreatment and the influence of pediatric PTSD. *Journal of Child and Adolescent Trauma, 7*(2), 121–130. doi:10.1007/s40653-014-0014-z

Kavanaugh, B., & Holler, K. (2015). Brief report: Neurocognitive functioning in adolescents following childhood maltreatment and evidence for underlying planning & organizational deficits. *Child Neuropsychologyeuropsychology, 21*(6), 840–848. doi:10.1080/09297049.2014.929101

Kavanaugh, B., Holler, K., & Selke, G. (2015). A neuropsychological profile of childhood maltreatment within an adolescent inpatient sample. *Applied Neuropsychology: Child, 4*, 9–19. doi:10.1080/21622965.2013.789964

Kirke-Smith, M., Henry, L., & Messer, D. (2014). Executive functioning: Developmental consequences on adolescents with histories of maltreatment. *British Journal of Developmental Psychology, 32*, 305–319. doi:10.1111/bjdp.12041

Kirke-Smith, M., Henry, L. A., & Messer, D. (2016). The effect of maltreatment type on adolescent executive functioning and inner speech. *Infant and Child Development, 25*, 516–532. doi:10.1002/icd.1951

Li, Y., Dong, F., Cao, F., Cui, N., Li, J., & Long, Z. (2013). Poly-victimization and executive functions in junior college students. *Scandinavian Journal of Psychology, 54*(6), 485–492. doi:10.1111/sjop.12083

Lim, L., Hart, H., Mehta, M. A., Simmons, A., Mirza, K., & Rubia, K. (2015). Neural correlates of error processing in young people with a history of severe childhood abuse: An fMRI study. *American Journal of Psychiatry, 172*(9), 892–900. doi:10.1176/appi.ajp.2015.14081042

Lind, T., Lee Raby, K., Caron, E. B., Roben, C. K. P., & Dozier, M. (2017). Enhancing executive functioning among toddlers in foster care with an attachment-based intervention. *Development and Psychopathology*, *29*, 575–586. doi:10.1017/S0954579417000190

McDermott, J. M., Westerlund, A., Zeanah, C. H., Nelson, C. A., & Fox, N. A. (2012). Early adversity and neural correlates of executive function: Implications for academic adjustment. *Developmental Cognitive Neuroscience, 2*, S59–S66. doi:10.1016/j.dcn.2011.09.008

Meguid, N., & Reda, M. (2016). Salivary cortisol levels in abused children with attention deficit hyperactivity disorder. *Journal of Psychiatry, 19*, 1–6. doi:10.4172/2378-5756.1000348

Mezzacappa, E., Kindlon, D. & Earls, F. (2001). Child abuse and performance task assessments of executive functions in boys. *Journal of Psychology and Psychiatry, 42*(8), 1041–1048. doi:10.1111/1469-7610.00803

Mothes, L., Kristensen, C. H., Grassi-Oliveira, R., Fonseca, R. P., de Lima Argimon, I. I., & Irigaray, T. Q. (2015). Childhood maltreatment and executive functions in adolescents. *Child and Adolescent Mental Health, 20*(1), 56–62. doi:10.1111/camh.12068

Mueller, S. C., Maheu, F. S., Dozier, M., Peloso, E., Mandell, D., Leibenluft, E., … Ernst, M. (2010). Early-life stress is associated with impairment in cognitive control in adolescence: An fMRI study. *Neuropsychologia, 48*(10), 3037–3044. doi:10.1016/j.neuropsychologia.2010.06.013

Nadeau, M. E., & Nolin, P. (2013). Attentional and executive functions in neglected children. *Journal of Child & Adolescent Trauma*, 6(1), 1–10. doi: 10.1080/19361521.2013.733794

Navalta, C. P., Polcari, A., Webster, D. M., Boghossian, A., & Teicher, M. T. (2006). Effects of childhood sexual abuse on neuropsychological and cognitive function in college women. *Journal of Neuropsychiatry and Clinical Neuroscience, 18*(1), 45–53. doi:10.1176/appi.neuropsych.18.1.45

Nolin, P., & Ethier, L. (2007). Using neuropsychological profiles to classify neglected children with or without physical abuse. *Child Abuse and Neglect, 31*(6), 631–643. doi:10.1016/j.chiabu.2006.12.009

Op den Kelder, R., Ensink, J. B. M., Overbeek, G., Maric, M., & Lindauer, R. J. L. (2017). Executive function as a mediator in the link between single or complex trauma and posttraumatic stress in children and adolescents. *Quality of Life Research, 26*(7), 1687–1696. doi:10.1007/s11136-017-1535-3

Park, S., Kim, B.-N., Choi, N.-H., Ryu, J., McDermott, B., Cobham, V., … Cho, S.-C. (2014). The effect of persistent posttraumatic stress disorder symptoms on executive functions in preadolescent children witnessing a single incident of death. *Anxiety, Stress & Coping: An International Journal, 27*, 241–252. doi:10.1080/10615806.2013.853049

Perna, R. B., & Kiefner, M. (2013). Long-term cognitive sequelae: Abused children without PTSD. *Applied Neuropsychology: Child, 2*(1), 1–5. doi:10.1080/09084282.2011.595460

Pollak, S. D., Nelson, C. A., Schlaak, M. F., Roeber, B. J., Wewerka, S. S., Wiik, K. L., … Gunnar, M. R. (2010). Neurodevelopmental effects of early deprivation in postinstitutionalized children. *Child Development, 81*(1), 224–236. doi:10.1111/j.1467-8624.2009.01391.x

Saigh, P. A, Yasik, A. E., Oberfield, R. a, Halamandaris, P. V, & Bremner, J. D. (2006). The intellectual performance of traumatized children and adolescents with or without posttraumatic stress disorder. *Journal of Abnormal Psychology, 115*(2), 332–340. doi:10.1037/0021-843X.115.2.332

Samuelson, K. W., Krueger, C. E., & Wilson, C. (2012). Relationships between maternal emotion regulation, parenting, and children’s executive functioning in families exposed to intimate partner violence. *Journal of Interpersonal Violence, 27*(17), 3532–3550. doi:10.1177/0886260512445385

Spann, M. N., Mayes, L. C., Kalmar, J. H., Guiney, J., Womer, F. Y., Pittman, B., … Blumberg, H. P. (2012). Childhood abuse and neglect and cognitive flexibility in adolescents. *Child Neuropsychologyeuropsychology, 18*(2), 182–189. doi:10.1080/09297049.2011.595400

Stewart, J. G., Kim, J. C., Esposito, E. C., Gold, J., Nock, M. K., & Auerbach, R. P. (2015). Predicting suicide attempts in depressed adolescents: Clarifying the role of disinhibition and childhood sexual abuse. *Journal of Affective Disorders, 187*, 27–34. doi:10.1016/j.jad.2015.08.034

Tibu, F., Sheridan, M. A., McLaughlin, K. A., Nelson, C. A., Fox, N. A., & Zeanah, C. H. (2016). Disruptions of working memory and inhibition mediate the association between exposure to institutionalization and symptoms of attention deficit hyperactivity disorder. *Psychological Medicine, 46*, 529–541. doi:10.1017/S0033291715002020

Tran, N. K., Van Berkel, S. R., van IJzendoorn, M. H., & Alink, L. R. A. (2017). The association between child maltreatment and emotional, cognitive, and physical health functioning in Vietnam. *BMC Public Health*, *17*, 1–13. doi:10.1186/s12889-017-4258-z

Valentino, K., Bridgett, D. J., Hayden, L. C., & Nuttall, A. K. (2012). Abuse, depressive symptoms, executive functioning, and overgeneral memory among a psychiatric sample of children and adolescents. *Journal of Clinical Child and Adolescent Psychology, 41*(4), 491–498. doi:10.1080/15374416.2012.660689

Viezel, K. D., Freer, B., & Lowell, A. (2015). Cognitive abilities of maltreated children. *Journal of Adolescence, 52*(1), 92–106. doi:10.1002/pits

Zou, Z., Meng, H., Ma, Z., Deng, W., Du, L., Wang, H., … Hu, H. (2013). Executive functioning deficits and childhood trauma in juvenile violent offenders in China. *Psychiatry Research, 207*, 218–224. doi:10.1016/j.psychres.2012.09.013
